# Supplementary material for: A game-factors approach to cognitive benefits from video-game training: A meta-analysis
Source: PLoS One. 2023 Aug 2;18(8):e0285925. doi: 10.1371/journal.pone.0285925 (PMC10395941; doi:10.1371/journal.pone.0285925)
Supplement: S1 Method — (DOCX) [file pone.0285925.s001.docx]

**S1 Method. Full Search Terms for Literature Search**

The full list of search terms utilized for the literature search are as follows: “Video Game Training”, “Videogame Training”, “Action Game Training”, “Strategy Game Training”, “Casual Game Training”, “Computer Game Training”, “Virtual Reality Training”, “VR Training”, “VR Cognitive Training”, “Video game learning”, “Videogame learning”, “Game Learning”, “Game Training”, “Action video game”, “Strategy video game”, “Casual video game”, “Computerized Cognitive Training”, “Computerized Working Memory Training”, “Computerized Memory Training”, “Brain Training”, “Game Cognition”, “Brain Age”, “Lumosity”, “Cogmed”, “Posit Science”, “Cognifit”, “Big Brain Academy”. Search terms for non-video-game cognitive intervention solutions (“Brain Age”, “Lumosity”, “Cogmed”, “Posit Science”, “Cognifit”, “Big Brain Academy”) were included to check for cases in which a video game control group was used against a non-game group of interest.
