# Supplementary material for: A game-factors approach to cognitive benefits from video-game training: A meta-analysis
Source: PLoS One. 2023 Aug 2;18(8):e0285925. doi: 10.1371/journal.pone.0285925 (PMC10395941; doi:10.1371/journal.pone.0285925)
Supplement: S1 Table — (DOCX) [file pone.0285925.s002.docx]

**S1 Table.** *Gameplay factors of games featured in included studies*

| *Game* | *Gameplay Footage Assessed* | *Genre (Action/Strategy)* | *Format (Long/Casual)* | *Movement (Ego, Allo)* | *Perspective (1st, 3rd)* | *Combat Game* | *Time Pressure* | *Multiple Object Control* | *Multiple Win States* | *Active Opponent* |
| --- | --- | --- | --- | --- | --- | --- | --- | --- | --- | --- |
| America's Army | <https://www.youtube.com/watch?v=iOzGI1oL1oc> | Act | L | E | 1^st^ | Y | Y | N | N | Y |
| Angry Birds | <https://www.youtube.com/watch?v=fdHtwXqLhGQ> | Str | C | A | 3^rd^ | N | N | N | N | N |
| Ballance | <https://www.youtube.com/watch?v=vxcilEBAdbI> | Str | L | E | 3^rd^ | N | Y | N | N | N |
| Battlezone | <https://www.youtube.com/watch?v=_Fff-1tFkpU> | Act | L | E | 3^rd^ | Y | Y | N | N | Y |
| Bejewelled 2 | <https://www.youtube.com/watch?v=tAu6abxsG1s> | Str | C | A | 3^rd^ | N | N | Y | N | N |
| Blockout | <https://www.youtube.com/watch?v=B2REJSeyfew> | Str | C | A | 3^rd^ | N | Y | Y | N | N |
| Boson-X | <https://www.youtube.com/watch?v=8zIMTVh_-0k> | Act | C | E | 3^rd^ | Y | N | N | N | N |
| Call of Duty 2 | <https://www.youtube.com/watch?v=AHRThPGp0Rs> | Act | L | E | 1^st^ | Y | Y | N | N | Y |
| Call of Duty 3 | <https://www.youtube.com/watch?v=QiOEkdODLBo> | Act | L | E | 1^st^ | Y | Y | N | N | Y |
| Call of Duty: Modern Warefare 3 DS | <https://www.youtube.com/watch?v=MYbkymmJnuc> | Act | L | E | 1^st^ | Y | Y | N | N | Y |
| Call of Duty 4: Modern Warfare | <https://www.youtube.com/watch?v=fNEbn1LfnAU> | Act | L | E | 1^st^ | Y | Y | N | N | Y |
| Call of Duty: Modern Warfare 2 | <https://www.youtube.com/watch?v=PPVq-pK2xk8> | Act | L | E | 1^st^ | Y | Y | N | N | Y |
| Call of Duty: Modern Warfare 3 | <https://www.youtube.com/watch?v=u3OCz9gikcY> | Act | L | E | 1^st^ | Y | Y | N | N | Y |
| Carmen Sandiego | <https://www.youtube.com/watch?v=--q8Hd-NLIY> | Str | C | A | 3^rd^ | N | N | N | N | N |
| Centipede | <https://www.youtube.com/watch?v=fUVgcfzIeGI> | Act | C | A | 3^rd^ | Y | Y | N | N | Y |
| Computer Solitaire | <https://www.youtube.com/watch?v=SZqnAfkyYqA> | Str | C | A | 3^rd^ | N | N | Y | N | N |
| Crazy Taxi | <https://www.youtube.com/watch?v=qjIGkv0_dEM> | Act | L | E | 3^rd^ | N | Y | N | Y | N |
| Cut the Rope | <https://www.youtube.com/watch?v=7HgssdcI-EM> | Str | C | A | 3^rd^ | N | N | Y | N | Y |
| Deer Hunter | <https://www.youtube.com/watch?v=kCehKGvvV4U> | Act | C | E | 1^st^ | Y | Y | N | N | Y |
| Donkey Kong | <https://www.youtube.com/watch?v=UBHJhla8TO4> | Act | C | A | 3^rd^ | N | Y | N | N | Y |
| EA Sports Active | <https://www.youtube.com/watch?v=B5staKDAfyU> | Str | L | A | 3^rd^ | N | Y | N | N | Y |
| FIFA 2010 | <https://www.youtube.com/watch?v=MzJTIoMcpqg> | Str | L | A | 3^rd^ | N | Y | Y | N | Y |
| FreeCell | <https://www.youtube.com/watch?v=udsWtRqUJAY> | Str | C | A | 3^rd^ | N | N | Y | N | N |
| Fruit Ninja | <https://www.youtube.com/watch?v=EvASnEgRMmM> | Act | C | E | 3^rd^ | N | Y | N | N | N |
| Fruit Ninja Kinect | https://www.youtube.com/watch?v=UTea43WWCyw | Act | C | E | 1st | N | Y | N | N | N |
| Fruit Ninja VR | https://www.youtube.com/watch?v=ZMpbTbq2acE | Act | C | E | 1st | N | Y | N | N | N |
| FTL: Faster Than Light | https://www.youtube.com/watch?v=vlW5I24DjGE | Act | L | A | 3rd | Y | Y | Y | N | Y |
| Ghost Recon 2 | <https://www.youtube.com/watch?v=y04jkYQAoRg> | Act | L | E | 1^st^ | Y | Y | N | N | Y |
| Half-Life 2 | <https://www.youtube.com/watch?v=aLbD1pD0JCk> | Act | L | E | 1^st^ | Y | Y | N | N | Y |
| Halo: Combat Evolved | <https://www.youtube.com/watch?v=J64Gn60y1o4> | Act | L | E | 1^st^ | Y | Y | N | N | Y |
| Harry Potter: Quidditch World Cup | <https://www.youtube.com/watch?v=axcszQi6DJM> | Act | L | E | 3^rd^ | N | Y | N | N | Y |
| Hidden Expedition-Everest | <https://www.youtube.com/watch?v=-b6hQX6sA6U> | Str | L | A | 3^rd^ | N | N | Y | N | N |
| Marble Madness | <https://www.youtube.com/watch?v=jazxSQz0yL4> | Act | L | E | 3^rd^ | N | Y | N | N | N |
| Mario Kart | <https://www.youtube.com/watch?v=AlAmXXNz5ac> | Act | L | E | 3^rd^ | N | Y | N | N | Y |
| Mario Kart DS | <https://www.youtube.com/watch?v=RB1QP8Zun7g> | Act | L | E | 3^rd^ | N | Y | N | N | Y |
| Medal of Honor | <https://www.youtube.com/watch?v=dbwfSRvzaLc> | Act | L | E | 1^st^ | Y | Y | N | N | Y |
| Medal of Honor: Allied Assault | <https://www.youtube.com/watch?v=lxF0hXJUqVc> | Act | L | E | 1^st^ | Y | Y | N | N | Y |
| Medal of Honor: Heroes 2 | <https://www.youtube.com/watch?v=n_5kInR9gQ4> | Act | L | E | 1^st^ | Y | Y | N | N | Y |
| Medal of Honor: Pacific Assault | <https://www.youtube.com/watch?v=UnVBP5qrxvI> | Act | L | E | 1^st^ | Y | Y | N | N | Y |
| Metal Gear Solid: Touch | https://www.youtube.com/watch?v=5zOOaPQ4hiE | Act | L | E | 3^rd^ | Y | Y | N | N | Y |
| Modern Combat: Sandstorm | <https://www.youtube.com/watch?v=kL0gsSMvZmg&index=10&list=PLC3DF80B6AA56088F> | Act | L | E | 1^st^ | Y | Y | N | N | Y |
| MultiTask* | http://multitaskgames.com/multitask-game.html | Act | L | A | 3^rd^ | N | Y | Y | N | N |
| Need for Speed | <https://www.youtube.com/watch?v=GScJzCQp81I> | Act | L | E | 3^rd^ | N | Y | N | N | Y |
| Pac-Man | <https://www.youtube.com/watch?v=dScq4P5gn4A> | Act | C | A | 3^rd^ | N | Y | N | N | Y |
| Pac-Man: Adventures in Time | <https://www.youtube.com/watch?v=uPGSLBsD2I4> | Act | C | A | 3^rd^ | N | Y | N | N | Y |
| Pinball Hall of Fame | <https://www.youtube.com/watch?v=NKT0Cl7_Uvk> | Act | L | A | 3^rd^ | N | Y | Y | Y | N |
| Portal | <https://www.youtube.com/watch?v=0P2dzIa6pZY> | Str | L | E | 1^st^ | N | Y | N | N | N |
| Portal 2 | https://www.youtube.com/watch?v=ZFqk8aj4-PA | Str | L | E | 1^st^ | N | Y | N | N | N |
| Red Dead Redemption | <https://www.youtube.com/watch?v=_7NzuoplXoY> | Act | L | E | 3^rd^ | Y | Y | N | Y | Y |
| Rise of Nations | <https://www.youtube.com/watch?v=aX-9N8BMtoQ> | Str | L | A | 3^rd^ | Y | Y | Y | Y | Y |
| Roller Coaster Tycoon III | <https://www.youtube.com/watch?v=yGsRAhGBChA> | Str | L | A | 3^rd^ | N | N | Y | Y | N |
| Smooth Snake* | http://snake.tap2play.io/ | Act | C | A | 3^rd^ | N | N | N | N | N |
| Starcraft II | <https://www.youtube.com/watch?v=X-5hcv-gUyM> | Str | L | A | 3^rd^ | Y | Y | Y | N | Y |
| Starctraft: Brood War | <https://www.youtube.com/watch?v=ukbE7hZSOn0&list=PLo6dUe-n7Er-0POryUIWuF0SB28Lso_LG> | Str | L | A | 3^rd^ | Y | Y | Y | N | Y |
| Starfront Collision | <https://www.youtube.com/watch?v=IqkdbCtFmiM> | Str | L | A | 3^rd^ | Y | Y | Y | N | Y |
| Star Wars Battlefront | https://www.youtube.com/watch?v=81YoEGhhjjM | Act | L | A | 3rd | Y | Y | N | N | Y |
| Super Mario 3D World | <https://www.youtube.com/watch?v=AxI_aQCRO2w> | Act | L | A | 3^rd^ | N | Y | N | N | Y |
| Super Mario 64 | https://www.youtube.com/watch?v=kGyhlf9XXlU | Act | L | E | 3^rd^ | N | Y | N | N | Y |
| Super Sniper | https://www.youtube.com/watch?v=Y0dZkA7EgfA | Act | C | E | 1^st^ | Y | Y | N | N | Y |
| Super Tetris | <https://www.youtube.com/watch?v=VaK7v8UNjo0> | Str | C | A | 3^rd^ | N | Y | Y | N | N |
| Tetris | <https://www.youtube.com/watch?v=oq0ApY0ooXM> | Str | C | A | 3^rd^ | N | Y | Y | N | N |
| The Sims | <https://www.youtube.com/watch?v=gi0AB5ksxuY> | Str | L | A | 3^rd^ | N | N | Y | Y | N |
| The Sims 2 | <https://www.youtube.com/watch?v=6qmgm-zpdKA> | Str | L | A | 3^rd^ | N | N | Y | Y | N |
| The Sims 3 | <https://www.youtube.com/watch?v=HPZRd-8s0n4> | Str | L | A | 3^rd^ | N | N | Y | Y | N |
| Unreal Tournament | <https://www.youtube.com/watch?v=4vHZcZJTegQ> | Act | L | E | 1^st^ | Y | Y | N | Y | Y |
| Unreal Tournament 2004 | <https://www.youtube.com/watch?v=AHRThPGp0Rs> | Act | L | E | 1^st^ | Y | Y | N | N | Y |
| Wii Fit Segway Circuit | <https://www.youtube.com/watch?v=ahfQAV18Jl0> | Act | L | E | 3^rd^ | N | Y | N | N | N |
| Word Whomp | <https://www.youtube.com/watch?v=kpyo1cKHQbM> | Str | C | A | 3^rd^ | N | N | Y | N | N |
| World of Warcraft | <https://www.youtube.com/watch?v=RMdYrALyPx4> | Str | L | E | 3^rd^ | Y | Y | N | Y | Y |
| Zaxxon | <https://www.youtube.com/watch?v=r8Ew5gMLulM> | Act | L | A | 3^rd^ | N | Y | N | N | Y |

*Note*.  *“Ego”* \ *“E”* = egocentric navigation. *“Allo”* \ *“A”* = allocentric navigation. *“Act”* = Action game. *“Str”*  = Strategy game. *“C”* = Casual game. *“L”* = Long-Form game *“1^st^”* = 1^st^-person perspective, *“3^rd^”* = 3^rd^-person perspective.

*Note.* * denotes games for which gameplay footage was not readily available. In these cases, direct links to those games (playable online) are provided

*Note.* Links included in this table were active and functional at the time the gameplay footage was assessed. We cannot guarantee that this will be the case at a future time.
