# Supplementary material for: A game-factors approach to cognitive benefits from video-game training: A meta-analysis
Source: PLoS One. 2023 Aug 2;18(8):e0285925. doi: 10.1371/journal.pone.0285925 (PMC10395941; doi:10.1371/journal.pone.0285925)
Supplement: S2 Table — (DOCX) [file pone.0285925.s003.docx]

**S2 Table.** *Outcome measures categorized by construct, with representative studies*

| Con. | Outcome Measure | Representative Study |
| --- | --- | --- |
| AP | 1-Back | Basak, Boot, Voss, & Kramer (2008) |
| AP | AFV 20o Accuracy | Wu & Spence (2013) |
| AP | Attention Blink | Belchiore, Yam, Thomas, Bavelier, Ball, Mann, & Marsiske (2019) |
| AP | Attention Network Test | Glass, Maddox, & Love (2013) |
| AP | Auditory Discrimination Overall RT | Green, Li, & Bavelier (2010) |
| AP | Auditory TOJ | Gonzales (2012) |
| AP | Card Rotation | Okagaki & Frensch (1994) |
| AP | Change Detection RT | Blacker, Curby, Klobusicksy, & Chein (2014) |
| AP | Change Localization IES | Momi, Smeralda, Sprugnoli, Neri, Rossi, Rossi… & Santarnecchi (2019) |
| AP | Choice Accuracy | Boot, Champion, Blakely, Wright, Souders, & Charness (2013) |
| AP | Choice RT | Boot, Champion, Blakely, Wright, Souders, & Charness (2013) |
| AP | Contrast Sensitivity Threshold - Total | Li, Polat, Scalzo, & Bavelier (2010) |
| AP | Crossmodal oddball - Alertness | Ballesteros, Mayas, Prieto, Ruiz-Marquez, Toril, & Reales (2017) |
| AP | Crossmodal oddball - Distraction | Ballesteros, Mayas, Prieto, Ruiz-Marquez, Toril, & Reales (2017) |
| AP | Crossmodal TOJ | Gonzales (2012) |
| AP | Cube Comparison | Okagaki & Frensch (1994) |
| AP | Digit Cancellation Task | Nouchi, Taki, Takeuchi, Hashizume, Akitsuki, Shigemune… & Kawashima (2012) |
| AP | Digit Symbol Substitution Task | Kűhn, Berna, Lűdtke, Gallinat, & Moritz (2018) |
| AP | Ennumeration | Boot, Kramer, Simons, Fabiani, & Gratton (2008) |
| AP | Ennumeration IES | Momi, Smeralda, Sprugnoli, Neri, Rossi, Rossi… & Santarnecchi (2019) |
| AP | Ennumeration Total Error | Green & Bavelier (2006 b) |
| AP | External Noise Interference Threshold | Bejjanki, Zhang, Li, Pouget, Green, Lu, & Bavelier (2014) |
| AP | Functional Field of View | Basak, Boot, Voss, & Kramer (2008) |
| AP | Figure Matching Acc | Nelson & Strachan (2009) |
| AP | Figure Matching RT | Nelson & Strachan (2009) |
| AP | Filter Task - Multi-Object Tracking | Oei & Patterson (2013) |
| AP | Form Board | Okagaki & Frensch (1994) |
| AP | Full Attention RT | Seçer & Satyen (2014) |
| AP | Global-Local Features - Global IES | Momi, Smeralda, Sprugnoli, Neri, Rossi, Rossi… & Santarnecchi (2019) |
| AP | Global-Local Features - Local IES | Momi, Smeralda, Sprugnoli, Neri, Rossi, Rossi… & Santarnecchi (2019) |
| AP | Go/No Go RT | McDermott (2013) |
| AP | Guilford-Zimmerman Visualization Task | Gagnon (1986) |
| AP | Interception | Adams (2013) |
| AP | Judgement of Line Orientation | Belchior, Marsiske, Sisco, Yam, Bavelier, Ball, & Mann (2013) |
| AP | Lateral masking threshold - total | Li, Polat, Scalzo, & Bavelier (2010) |
| AP | Lexical Decision Nonwords RT | van Ravenzwaaij, Boekel, Fortsmann, Ratcliff, & Wagenmakers (2014) |
| AP | Lexical Decision Words RT | van Ravenzwaaij, Boekel, Fortsmann, Ratcliff, & Wagenmakers (2014) |
| AP | Location Task Acc | Nelson & Strachan (2009) |
| AP | Location Task RT | Nelson & Strachan (2009) |
| AP | Manikin | Kűhn, Berna, Lűdtke, Gallinat, & Moritz (2018) |
| AP | MATB System Monitoring Timeout Errors | Chiappe, Conger, Liao, Caldwell, & Vu (2013) |
| AP | Mental Rotation - RT | Basak, Boot, Voss, & Kramer (2008) |
| AP | Moving Dots RT | van Ravenzwaaij, Boekel, Fortsmann, Ratcliff, & Wagenmakers (2014) |
| AP | Multiple Object Tracking | Belchior, Yam, Thomas, Bavelier, Ball, Mann, & Marsiske (2019) |
| AP | Number Comparisons | Perrot, Maillot, & Hartley (2019) |
| AP | Object Rotation | Belchior, Yam, Thomas, Bavelier, Ball, Mann, & Marsiske (2019) |
| AP | Oddball - Alertness | Ruiz-Marquez, Prieto, Mayas, Toril, Reales, & Ballesteros (2019) |
| AP | Perceptual Speed | Okagaki & Frensch (1994) |
| AP | Race RT | Adams (2013) |
| AP | Repetition RT | Colzato, van den Wildenberg, & Hommel (2019) |
| AP | RMS Error | Li, Chen, & Chen (2016) |
| AP | Road Sign Test | Belchior, Yam, Thomas, Bavelier, Ball, Mann, & Marsiske (2019) |
| AP | Rochester UFOV | Belchior, Yam, Thomas, Bavelier, Ball, Mann, & Marsiske (2019) |
| AP | Serial Reaction Time IES | Momi, Smeralda, Sprugnoli, Neri, Rossi, Rossi… & Santarnecchi (2019) |
| AP | Simple Accuracy | Boot, Champion, Blakely, Wright, Souders, & Charness (2013) |
| AP | Simple Reaction Time | Nouchi, Taki, Takeuchi, Hashizume, Akitsuki, Shigemune… & Kawashima (2012) |
| AP | Spatial Errors Standardized MEasure | Subrahmanyam & Greenfield (1994) |
| AP | Spatial Orintation Task | Shute, Ventura, & Ke (2015) |
| AP | Spatial Relations Test | Dorval & Pepin (1986) |
| AP | Speed of Processing Task - Animals | Minear, Brasher, Guerror, Brasher, Moore, & Sukeena (2016) |
| AP | Speed of Processing Task - Array | Minear, Brasher, Guerror, Brasher, Moore, & Sukeena (2016) |
| AP | Speed of Processing Task - Dot | Minear, Brasher, Guerror, Brasher, Moore, & Sukeena (2016) |
| AP | Speed of Processing Task - Words | Minear, Brasher, Guerror, Brasher, Moore, & Sukeena (2016) |
| AP | Stopping Task - Go RT | Basak, Boot, Voss, & Kramer (2008) |
| AP | Symbol Search | Nouchi, Taki, Takeuchi, Hashizume, Akitsuki, Shigemune… & Kawashima (2012) |
| AP | Task Switching - Non-Switch RT | Basak, Boot, Voss, & Kramer (2008) |
| AP | ToVA - Iconic Memory Buffer | Schubert, Finke, Redel, Kluckow, Müller, & Strobach (2015) |
| AP | ToVA - lateral spatial distribution | Schubert, Finke, Redel, Kluckow, Müller, & Strobach (2015) |
| AP | ToVA - Processing Speed | Schubert, Finke, Redel, Kluckow, Müller, & Strobach (2015) |
| AP | ToVA - Sensory Processing | Schubert, Finke, Redel, Kluckow, Müller, & Strobach (2015) |
| AP | ToVA - vertical spatial distribution | Schubert, Finke, Redel, Kluckow, Müller, & Strobach (2015) |
| AP | Trails A | McCord, Cocks, Barreiros, & Bizo (2020) |
| AP | UAB UFOV | Belchior, Yam, Thomas, Bavelier, Ball, Mann, & Marsiske (2019) |
| AP | UFOV | Feng, Spence, & Pratt (2007) |
| AP | Visual Discrimination Overall RT | Green, Li, & Bavelier (2010) |
| AP | Visual Search | Glass, Maddox, & Love (2013) |
| AP | Visual TOJ | Gonzales (2012) |
| HC | 2-Back Accuracy | Basak, Boot, Voss, & Kramer (2008) |
| HC | Alignment Span | Minear, Brasher, Guerror, Brasher, Moore, & Sukeena (2016) |
| HC | Alternation RT | Colzato, van den Wildenberg, & Hommel (2019) |
| HC | Alternation RT | Colzato, van den Wildenberg, & Hommel (2019) |
| HC | Aritmetic | Nouchi, Taki, Takeuchi, Hashizume, Akitsuki, Shigemune… & Kawashima (2012) |
| HC | Aritmetic Aptitude | Minear, Brasher, Guerror, Brasher, Moore, & Sukeena (2016) |
| HC | Attention Network Task -EA | Minear, Brasher, Guerror, Brasher, Moore, & Sukeena (2016) |
| HC | Backward Digit Span | McCord, Cocks, Barreiros, & Bizo (2020) |
| HC | Backward Spatial Span | Nouchi, Taki, Takeuchi, Hashizume, Akitsuki, Shigemune… & Kawashima (2012) |
| HC | Berg Card Sorting Test - Correct Responses | Martincevic & Vranic (2020) |
| HC | Berg Card Sorting Test - Errors | Martincevic & Vranic (2020) |
| HC | Bivalen Shapest Test - Cost | Martincevic & Vranic (2020) |
| HC | Cattell | Minear, Brasher, Guerror, Brasher, Moore, & Sukeena (2016) |
| HC | Cognitive Load | Novak & Tassell (2015) |
| HC | Color-Word Stroop Accuracy | Whitlock, McLaughlin, & Allaire (2012) |
| HC | Complex Span | Oei & Patterson (2013) |
| HC | Corsi Block Tapping | Martincevic & Vranic (2020) |
| HC | Delayed Matching-To-Sample Task | Strenziok, Parasuraman, Clarke, Cisler, Thompson, & Greenwood (2014) |
| HC | Digit Cancellation TaSk | Nouchi, Taki, Takeuchi, Hashizume, Akitsuki, Shigemune… & Kawashima (2012) |
| HC | Divided minus Full Attention RT | Seçer & Satyen (2014) |
| HC | Dual n-back Task | Martincevic & Vranic (2020) |
| HC | ECB Reasoning | Boot, Champion, Blakely, Wright, Souders, & Charness (2013) |
| HC | Filter Task - Cognitive Control | Oei & Patterson (2013) |
| HC | Flanker Task | Oei & Patterson (2014 a) |
| HC | Frontal Assesment at Bedside | Nouchi, Taki, Takeuchi, Hashizume, Akitsuki, Shigemune… & Kawashima (2012) |
| HC | Go/No Go | Oei & Patterson (2014 a) |
| HC | Information Filtering | Glass, Maddox, & Love (2013) |
| HC | Insight | Shute, Ventura, & Ke (2015) |
| HC | Inteferences | Minear, Brasher, Guerror, Brasher, Moore, & Sukeena (2016) |
| HC | Japanese Reading Test | Nouchi, Taki, Takeuchi, Hashizume, Akitsuki, Shigemune… & Kawashima (2012) |
| HC | Kaufman Brief Intelligence Test | Valdez & Ferguson (2012) |
| HC | Letter No-Go IES | Momi, Smeralda, Sprugnoli, Neri, Rossi, Rossi… & Santarnecchi (2019) |
| HC | Letter Sets | Boot, Champion, Blakely, Wright, Souders, & Charness (2013) |
| HC | Letter-Number Sequencing | McCord, Cocks, Barreiros, & Bizo (2020) |
| HC | Letter-Number Span | Minear, Brasher, Guerror, Brasher, Moore, & Sukeena (2016) |
| HC | Mathematics Aptitude | Minear, Brasher, Guerror, Brasher, Moore, & Sukeena (2016) |
| HC | Multi-Location Switching | Glass, Maddox, & Love (2013) |
| HC | Nback - ACC | Boot, Kramer, Simons, Fabiani, & Gratton (2008) |
| HC | N-Back Hits-FA | Ballesteros, Mayas, Prieto, Ruiz-Marquez, Toril, & Reales (2017) |
| HC | N-back Memory Load Cost | Basak, Boot, Voss, & Kramer (2008) |
| HC | Nonsense Syllogisms | Minear, Brasher, Guerror, Brasher, Moore, & Sukeena (2016) |
| HC | Object N-Back | Minear, Brasher, Guerror, Brasher, Moore, & Sukeena (2016) |
| HC | Oddball - Distraction | Ruiz-Marquez, Prieto, Mayas, Toril, Reales, & Ballesteros (2019) |
| HC | Operation Span | Basak, Boot, Voss, & Kramer (2008) |
| HC | Paper Folding | Sanchez (2012) |
| HC | Plus-Minus Task Cost | Martincevic & Vranic (2020) |
| HC | Ravens Matricies | Basak, Boot, Voss, & Kramer (2008) |
| HC | Reading Span | Minear, Brasher, Guerror, Brasher, Moore, & Sukeena (2016) |
| HC | Remote Association Task | Shute, Ventura, & Ke (2015) |
| HC | Remote Association Task - Problem Solving | Shute, Ventura, & Ke (2015) |
| HC | Response Selection Tast - Incompatible | Clarke, Lanphear, & Riddick (1987) |
| HC | Reverse Striio Task | Nouchi, Taki, Takeuchi, Hashizume, Akitsuki, Shigemune… & Kawashima (2012) |
| HC | Rotation Span | Minear, Brasher, Guerror, Brasher, Moore, & Sukeena (2016) |
| HC | Simon task | Hutchinson, Barrett, Nitka, & Raynes (2015) |
| HC | Stop Probability | Basak, Boot, Voss, & Kramer (2008) |
| HC | Stroop Task | Nouchi, Taki, Takeuchi, Hashizume, Akitsuki, Shigemune… & Kawashima (2012) |
| HC | Switch Cost | Green, Sugarman, Medford, Klobusicky, & Bavelier (2012) |
| HC | Symmetry Span | Minear, Brasher, Guerror, Brasher, Moore, & Sukeena (2016) |
| HC | Task Switch - Total Switch Cost | Boot, Kramer, Simons, Fabiani, & Gratton (2008) |
| HC | Task Switching | Glass, Maddox, & Love (2013) |
| HC | Task Switching Task | Oei & Patterson (2014 a) |
| HC | Total Math Score | Novak & Tassell (2015) |
| HC | ToVA - top-down control | Schubert, Finke, Redel, Kluckow, Müller, & Strobach (2015) |
| HC | Tower of London | Boot, Kramer, Simons, Fabiani, & Gratton (2008) |
| HC | Trail Making A+B | Kűhn, Berna, Lűdtke, Gallinat, & Moritz (2018) |
| HC | Trail Making B-A | Perrot, Maillot, & Hartley (2019) |
| HC | Trail Making Task part B | Nouchi, Taki, Takeuchi, Hashizume, Akitsuki, Shigemune… & Kawashima (2012) |
| HC | Trails B | McCord, Cocks, Barreiros, & Bizo (2020) |
| HC | Verbal N-Back | Minear, Brasher, Guerror, Brasher, Moore, & Sukeena (2016) |
| HC | Visual Search - Dual - Total RT | Oei & Patterson (2013) |
| HC | WAIS-III Matrix Reasoning | Strenziok, Parasuraman, Clarke, Cisler, Thompson, & Greenwood (2014) |
| HC | Wisconson Card Sorting Task | Nouchi, Taki, Takeuchi, Hashizume, Akitsuki, Shigemune… & Kawashima (2012) |
| Mem | Arrow Span | Minear, Brasher, Guerror, Brasher, Moore, & Sukeena (2016) |
| Mem | Block Design | Belchior, Yam, Thomas, Bavelier, Ball, Mann, & Marsiske (2019) |
| Mem | Circle Span | Minear, Brasher, Guerror, Brasher, Moore, & Sukeena (2016) |
| Mem | ECB Recognition | Boot, Champion, Blakely, Wright, Souders, & Charness (2013) |
| Mem | Foreward Digit Span | McDermott (2013) |
| Mem | Forwatd Digit Spand | Nouchi, Taki, Takeuchi, Hashizume, Akitsuki, Shigemune… & Kawashima (2012) |
| Mem | Letter Recall | Novak & Tassell (2015) |
| Mem | Letter Span | Minear, Brasher, Guerror, Brasher, Moore, & Sukeena (2016) |
| Mem | Meanigful Memory | Boot, Champion, Blakely, Wright, Souders, & Charness (2013) |
| Mem | MSEQ | Boot, Champion, Blakely, Wright, Souders, & Charness (2013) |
| Mem | MST-LDI | Clemenson, & Stark (2015) |
| Mem | MST-Recognition | Clemenson, & Stark (2015) |
| Mem | Multiple Object Tracking - Capacity | Boot, Kramer, Simons, Fabiani, & Gratton (2008) |
| Mem | Sakai Visual Short-Term Memory Task | McDermott (2013) |
| Mem | Spatial Relations | Kűhn, Berna, Lűdtke, Gallinat, & Moritz (2018) |
| Mem | Sternberg Visual Memory Test (RT) | Goldstein, Cajko, Oosterbroek, Michielsen, van Hauten, & Saverda (1997) |
| Mem | ToVA - Storage Capacity | Schubert, Finke, Redel, Kluckow, Müller, & Strobach (2015) |
| Mem | Virtual Spatial navigation Assesment | Shute, Ventura, & Ke (2015) |
| Mem | Visual Memory Total | Oei & Patterson (2013) |
| Mem | VNSA - Spatial Ability | Shute, Ventura, & Ke (2015) |
| Mem | VSTM Task | Boot, Kramer, Simons, Fabiani, & Gratton (2008) |
| Mem | WAIS-III Logical Memory | Strenziok, Parasuraman, Clarke, Cisler, Thompson, & Greenwood (2014) |
| PS | Affective RT (Angry) | Bailey & West (2013) |
| PS | Balloon Analoge Risk Task | Glass, Maddox, & Love (2013) |
| PS | Beck Depression Inventory | Valdez & Ferguson (2012) |
| PS | Confidence | Novak & Tassell (2015) |
| PS | Geriatric depression | Belchior, Yam, Thomas, Bavelier, Ball, Mann, & Marsiske (2019) |
| PS | Affective RT (Happy) | Bailey & West (2013) |
| PS | Mathematics Anxiety | Novak & Tassell (2015) |
| PS | MMSE | Nouchi, Taki, Takeuchi, Hashizume, Akitsuki, Shigemune… & Kawashima (2012) |
| PS | Neutral RT | Bailey & West (2013) |
| PS | Older Person's Quality of Life Composite Score | McCord, Cocks, Barreiros, & Bizo (2020) |
| PS | PANAS negative affect | Belchior, Yam, Thomas, Bavelier, Ball, Mann, & Marsiske (2019) |
| PS | PANAS positive affect | Belchior, Yam, Thomas, Bavelier, Ball, Mann, & Marsiske (2019) |
| PS | Preparing to Overcome Prepotency | Momi, Smeralda, Sprugnoli, Neri, Rossi, Rossi… & Santarnecchi (2019) |
| PS | Sandia IES | Momi, Smeralda, Sprugnoli, Neri, Rossi, Rossi… & Santarnecchi (2019) |
| PS | State Hostility Scale | Valdez & Ferguson (2012) |
| PS | TIALD Accuracy | Belchior, Yam, Thomas, Bavelier, Ball, Mann, & Marsiske (2019) |

*Note.* Con = Construct. AP = Attention & Perception construct. HC = Higher-order Cognition construction. Mem = Memory construct. PS = Psychosocial construct.
