# Supplementary material for: A game-factors approach to cognitive benefits from video-game training: A meta-analysis
Source: PLoS One. 2023 Aug 2;18(8):e0285925. doi: 10.1371/journal.pone.0285925 (PMC10395941; doi:10.1371/journal.pone.0285925)
Supplement: S3 Table — (DOCX) [file pone.0285925.s004.docx]

**S3 Table.** *Characteristics of included video-game training studies*

| *Citation* | *Group/Condition* | *N* | *Standardized Mean Gain (ES_sg_*) | *SE of the ES_sg_* | *Weight (w_sg_)* | *Ave. Age* | *% Female* | *Duration (Hrs)* | | *Active Control* | *Is Control* | | *Game Used* |  |
| --- | --- | --- | --- | --- | --- | --- | --- | --- | --- | --- | --- | --- | --- | --- |
| Adams (2013) | Expt 3a, Action Game Group | 10 | 1.01 | 0.38 | 6.78 | 20.13 | 100 | | 1 | Y | N | *Unreal Tournament 2004* | | |
|  | Expt 3a, Non-Action Game Group | 10 | 1.13 | 0.39 | 6.6 | 20.13 | 100 | | 1 | Y | Y | *Tetris* | | |
| Bailey & West (2013) | Action Game Group | 10 | 2.47 | 0.64 | 2.48 | 21.77 | 0 | | 10 | Y | N | *Unreal Tournament 3* | | |
|  | Non-Action Game Group | 9 | 2.59 | 0.70 | 2.07 | 20.4 | 0 | | 10 | Y | N | *Tetris* | | |
| Ballesteros, Mayas, Prieto, Ruiz-Marquez, Toril, & Reales (2017) | Active Control Group | 25 | 0.18 | 0.20 | 24.6 | 64.52 | 50 | | 12 | Y | Y | *The Sims* | | |
| Basak, Boot, Voss, & Kramer (2008) | Experimental Group | 19 | 0.25 | 0.23 | 18.29 | 74.1 | 68.75 | | 24 | N | N | *Rise of Nations* | | |
| Bejjanki, Zhang, Li, Pouget, Green, Lu, & Bavelier (2014) | Expt. 2 Action Game Group | 12 | 0.74 | 0.33 | 9.43 | 23.9 | 25 | | 50 | Y | N | *Unreal Tournament 2004* | | |
|  | Expt. 2 Control group | 14 | 0.05 | 0.27 | 13.98 | 22.6 | 64.29 | | 50 | Y | Y | *The Sims 2* | | |
| Belchior, Marsiske, Sisco, Yam, Bavelier, Ball, & Mann (2013) | Experimental Group | 14 | 0.32 | 0.27 | 13.3 | 74.5 | 51.72 | | 9 | Y | N | *Medal of Honor* | | |
|  | Placebo Control Group | 15 | 0.38 | 1.80 | 0.31 | 74.5 | 52.72 | | 9 | Y | Y | *Tetris* | | |
| Belchior, Yam, Thomas, Bavelier, Ball, Mann, & Marsiske (2019) | Videogame CT | 17 | 0.25 | 0.25 | 16.47 | 73.2 | 63 | | 60 | Y | N | *Crazy Taxy* | | |
| Blacker, Curby, Klobusicksy, & Chein (2014) | "Action Game" Condition | 17 | 1.21 | 0.32 | 9.82 | 20.41 | 50 | | 30 | Y | N | *Call of Duty: Modern Warfare 3* | | |
|  | Control Condition | 17 | 0.06 | 0.24 | 16.7 | 20.65 | 50 | | 30 | Y | Y | *The Sims 3* | | |
| Boot, Champion, Blakely, Wright, Souders, & Charness (2013) | "Action Game" Group | 14 | -0.34 | 0.28 | 16.41 | 75 | 50 | | 60 | Y | N | *Mario Kart DS* | | |
| Boot, Kramer, Simons, Fabiani, & Gratton (2008) | MOH Group | 20 | 0.52 | 0.25 | 17.27 | 21.35 | 90 | | 22.5 | Y | N | *Medal of Honor* | | |
|  | RON Group | 20 | 0.46 | 0.24 | 16.89 | 21.74 | 85 | | 22.5 | Y | N | *Rise of Nations* | | |
|  | Tetris Group | 20 | 0.36 | 0.24 | 12.43 | 21.5 | 90 | | 22.5 | Y | N | *Tetris* | | |
| Cherney, Bersted, & Smetter (2014) | Gamecube Condition | 20 | 1.21 | 0.29 | 11.58 | 20.5 | 50 | | 1 | Y | N | *Crazy Taxi* | | |
|  | Wii Condition | 20 | 1.35 | 0.31 | 10.44 | 20.5 | 50 | | 1 | Y | N | *Wii Fit Segway Circuit* | | |
| Chiappe, Conger, Liao, Caldwell, & Vu (2013) | Experimental Group | 26 | 0.68 | 0.22 | 21.14 | 22 | 50 | | 50 | N | N | *Ghost Recon 2* | | |
| Clarke, Lanphear, & Riddick (1987) | Experimental Group | 14 | 0.67 | 0.30 | 11.45 | 70 | 57.14 | | 14 | N | N | *Donkey Kong, Pac-Man* | | |
| Clemenson, & Stark (2015) | Expt. 2, Active Control Group | 22 | 0.09 | 0.21 | 21.91 | 20 | 63.64 | | 5 | Y | Y | *Angry Birds* | | |
|  | Expt. 2, Experimental Group | 23 | 0.17 | 0.21 | 22.66 | 20 | 73.91 | | 5 | Y | N | *Super Mario 3D World* | | |
| Cohen, Green, & Bavelier (2008) | Control Group #1* | 14 | 4.90 | 0.96 | 1.08 | 19.9 | 42.86 | | 12 | Y | Y | *America's Army* | | |
|  | Control Group #2* | 14 | 9.05 | 1.73 | 0.34 | 20.1 | 50 | | 12 | Y | Y | *Harry Potter: Quidditch World Cup* | | |
|  | Control Group #3* | 14 | 4.74 | 0.93 | 1.148 | 19.5 | 42.86 | | 12 | Y | Y | *Tetris* | | |
|  | Experimental Group | 11 | 3.24 | 0.75 | 1.768 | 21.6 | 45.45 | | 12 | Y | N | *Unreal Tournament 2004* | | |
| Colzato, van den Wildenberg, & Hommel (2019) | Met Carriers group* | 38 | 3.81 | 0.47 | 4.6 | 22.1 | 53 | | 10 | N | N | *Half-Life 2* | | |
|  | Homozygous Group* | 56 | 4.54 | 0.45 | 4.95 | 21.3 | 42 | | 10 | N | N | *Half-Life 2* | | |
| De Lisi & Cammarano (1996) | Blockout condition | 57 | 0.59 | 0.14 | 1.76 | 21.5 | 73.68 | | 1 | Y | N | *Blockout* | | |
|  | Solitaire condition | 53 | 0.40 | 0.14 | 48.47 | 21.5 | 79.24 | | 1 | Y | Y | *Computer Solitaire* | | |
| De Lisi & Wolfard (2002) | Control Group | 24 | -0.01 | 0.20 | 49.13 | 8.5 | 50 | | 5.5 | Y | Y | *Carmen Sandiego* | | |
|  | Experimental Group | 23 | 1.18 | 0.27 | 24 | 8.5 | 47.8 | | 5.5 | Y | N | *Tetris* | | |
| Dorval & Pepin (1986) | Experimental Group | 38 | 0.57 | 0.17 | 13.52 | 22 | 50 | | 6.67 | N | N | *Zaxxon* | | |
| Feng, Spence, & Pratt (2007) | Expt. 2, Control Condition, Females | 7 | 0.60 | 0.41 | 32.68 | 25 | 100 | | 10 | Y | N | *Ballance* | | |
|  | Expt. 2, Control Condition, Males | 3 | -0.03 | 0.58 | 5.93 | 25 | 0 | | 10 | Y | Y | *Ballance* | | |
|  | Expt. 2, Experimental Condition, Females | 7 | 1.69 | 0.59 | 3 | 25 | 100 | | 10 | Y | N | *Medal of Honor: Pacific Assault* | | |
|  | Expt. 2, Experimental condition, Males | 3 | 1.55 | 0.86 | 2.88 | 25 | 0 | | 10 | Y | Y | *Medal of Honor: Pacific Assault* | | |
| Gagnon (1986) | Interactive Treatment Condition | 30 | 0.45 | 0.19 | 1.36 | 28 | 100 | | 0.5 | N | N | *Battlezone* | | |
| Glass, Maddox, & Love (2013) | "SC1" Condition | 26 | 2.01 | 0.44 | 27.24 | 20.3 | 50 | | 40 | Y | N | *Starcraft: Brood War* | | |
|  | "SC2" Condition | 26 | 2.29 | 0.44 | 5.27 | 20.4 | 50 | | 40 | Y | N | *Starcraft II* | | |
|  | "Sims" Condition* | 26 | 4.30 | 1.19 | 5.2 | 19.9 | 50 | | 40 | Y | Y | *The Sims* | | |
| Goldstein, Cajko, Oosterbroek, Michielsen, van Hauten, & Saverda (1997) | Experimental Group | 10 | 0.95 | 0.41 | 5.98 | 76.5 | 50 | | 30.5 | N | N | *Super Tetris* | | |
| Gonzales (2012) | Expt. 4-6, Action Game Group | 12 | 0.49 | 0.31 | 10.7 | 22 | 80 | | 10 | Y | N | *Call of Duty 4: Modern Warfare* | | |
|  | Expt. 4-6, Non-Action Game Group | 13 | 0.16 | 0.28 | 12.84 | 23 | 76.92 | | 10 | Y | Y | *Pinball Hall of Fame* | | |
| Green & Bavelier (2006 b) | Expt. 2, Control Group | 8 | 0.04 | 0.30 | 11.07 | 19.7 | 50 | | 10 | Y | Y | *Tetris* | | |
|  | Expt. 2, Experimental Group | 9 | 0.21 | 0.30 | 11.28 | 20.4 | 55.56 | | 10 | Y | N | *Medal of Honor: Allied Assault* | | |
| Green, Li, & Bavelier (2010) | Expt. 3, Control Group | 11 | 0.14 | 0.30 | 10.89 | 24.7 | 63.63 | | 50 | Y | Y | *The Sims 2* | | |
|  | Expt. 3, Experimental Group | 11 | 0.16 | 0.30 | 10.86 | 25.7 | 50 | | 50 | Y | N | *Unreal Tournament 2004, Call of Duty 2* | | |
| Green, Sugarman, Medford, Klobusicky, & Bavelier (2012) | Expt. 4 action | 18 | 0.00 | 0.24 | 18 | 25.7 | 61 | | 50 | Y | N | *Unreal Tournament 2004* | | |
|  | Expt. 4 control | 17 | 3.03 | 0.57 | 3.03 | 24.7 | 76 | | 50 | Y | Y | *The Sims 2* | | |
| Huang (2020) | Immersive Group | 16 | 0.40 | 0.49 | 4.2 | 61.93 | 72.7 | | 2.67 | Y | N | *Fruit Ninja VR* | | |
|  | Non-Immersive Group | 16 | 0.74 | 0.55 | 3.3 | 61.93 | 72.7 | | 2.67 | Y | Y | *Fruit Ninja Kinect* | | |
| Hutchinson, Barrett, Nitka, & Raynes (2015) | Call of Duty DS group | 14 | 1.95 | 0.46 | 4.82 | 21.5 | 90 | | 10 | Y | N | *Call of Duty 3 Modern Warefare 3 DS* | | |
|  | Call of Duty group | 10 | 2.86 | 0.71 | 1.96 | 21.5 | 85.71 | | 10 | Y | N | *Call of Duty 3* | | |
| Kűhn, Berna, Lűdtke, Gallinat, & Moritz (2018) | Experimental Group | 21 | 0.15 | 0.22 | 20.78 | 45 | 52 | | ** | N | N | *Boson-X* | | |
| Li, Chen, & Chen (2016) | Expt. 3 Action group | 6 | 2.14 | 0.74 | 1.83 | 24 | 50 | | 10 | Y | N | *Mario Kart* | | |
|  | Expt. 3 Control Group | 6 | 1.29 | 0.55 | 3.27 | 22 | 50 | | 10 | Y | Y | *Roller Coaster Tycoon III* | | |
| Li, Polat, Scalzo, & Bavelier (2010) | Expt. 2, Action Group | 6 | 0.42 | 0.43 | 5.52 | 26.51 | 50 | | 50 | Y | N | *Unreal Tournament 2004* | | |
|  | Expt. 3, Action Group | 14 | 0.19 | 0.27 | 6.38 | 26 | 50 | | 50 | Y | N | *Unreal Tournament 2004* | | |
|  | Expt. 2, Non-Action Group | 7 | 0.44 | 0.40 | 5.69 | 24.9 | 28.57 | | 50 | Y | Y | *The Sims 2* | | |
|  | Expt. 3, Non-Action Group | 11 | -0.04 | 0.30 | 6.79 | 24.7 | 63.64 | | 50 | Y | Y | *The Sims 2* | | |
| Martincevic & Vranic (2020) | Multitasking Group | 37 | 0.28 | 0.42 | 1.85 | 20.46 | 76 | | 4 | Y | N | *MultiTask* | | |
|  | Active Control Group | 37 | 0.29 | 0.38 | 14.8 | 20.22 | 72 | | 4 | Y | Y | *Smooth Snake* | | |
| McCord, Cocks, Barreiros, & Bizo (2020) | Experimental Group | 12 | .29 | .73 | 31.41 | 89.5 | 83.3 | | 3 | Y | N | *Star Wars Battlefront* | | |
| McDermott (2013) | "Action Games" Condition | 15 | 0.10 | 0.26 | 7.83 | 68.9 | 80 | | 50 | Y | N | *Medal of Honor: Heroes 2* | | |
| Minear, Brasher, Guerror, Brasher, Moore, & Sukeena (2016) | "Video Game" Condition | 33 | 0.23 | 0.18 | 9.92 | 19.6 | 45.45 | | 18 | Y | N | *Starcraft: Brood War* | | |
| Momi, Smeralda, Sprugnoli, Neri, Rossi, Rossi… & Santarnecchi (2019) | Experimental Group | 25 | 2.09 | 0.36 | 9.34 | 24.2 | 36 | | 30 | N | N | *CounterStrike: Global Offensive* | | |
| Nelson & Strachan (2009) | Expt. 1, Action group | 10 | 0.13 | 0.32 | 11.07 | 21.8 | 50 | | 1 | Y | N | *Unreal Tournament* | | |
|  | Expt. 1, Puzzle group | 10 | 0.14 | 0.33 | 11.28 | 21.8 | 40 | | 1 | Y | N | *Portal* | | |
| Nouchi, Taki, Takeuchi, Hashizume, Akitsuki, Shigemune… & Kawashima (2012) | Tetris group | 14 | 0.08 | 0.27 | 13.67 | 69.13 | 50 | | 20 | Y | Y | *Tetris* | | |
| Nouchi, Kawata, Saito, Hummelmeier, Nakamura… & Kawashima (2020) | Active Control group | 36 | 0.27 | 0.54 | 3.37 | 21.63 | 66.7 | | 9.33 | Y | Y | *Tetris* | | |
| Novak & Tassell (2015) | Expt. 2, Action Game group | 14 | 0.11 | 0.28 | 12.63 | 19.64 | 92.86 | | 10 | Y | N | *Unreal Tournament 2004* | | |
|  | Expt. 2, Active Control Group | 16 | 0.18 | 0.26 | 15.08 | 19.64 | 93.75 | | 10 | Y | Y | *Angry Birds* | | |
| Oei & Patterson (2013) | "Bejeweled" Condition | 15 | 0.43 | 0.27 | 13.66 | 21.07 | 37.33 | | 20 | Y | N | *Bejewelled 2* | | |
|  | "Hidden Object" Condition | 14 | 0.27 | 0.28 | 13.21 | 21.07 | 37.33 | | 20 | Y | N | *Hidden Expedition-Everest* | | |
|  | "Modern Combat" Condition | 16 | 0.68 | 0.27 | 13.36 | 21.07 | 37.33 | | 20 | Y | N | *Modern Combat: Sandstorm* | | |
|  | "The Sims" Condition | 16 | 0.22 | 0.26 | 14.97 | 21.07 | 37.33 | | 20 | Y | N | *The Sims 3* | | |
| Oei & Patterson (2014a) | Cut the Rope group | 14 | 1.19 | 0.35 | 8.18 | 21.06 | 44.23 | | 20 | Y | N | *Cut the Rope* | | |
|  | Fruit Ninja group | 14 | 0.20 | 0.27 | 13.72 | 21.06 | 44.23 | | 20 | Y | N | *Fruit Ninja* | | |
|  | Modern Combat group | 10 | 0.38 | 0.33 | 9.32 | 21.06 | 44.23 | | 20 | Y | N | *Modern Combat: Sandstorm* | | |
|  | Sarfront Collision group | 14 | 0.41 | 0.28 | 12.91 | 21.06 | 44.23 | | 20 | Y | N | *Starfront Collision* | | |
| Oei & Patterson (2015) | Modern Combat Group | 13 | 1.88 | 0.46 | 4.71 | 21.78 | 55 | | 20 | Y | N | *Modern Combat: Sandstorm* | | |
|  | MGS Touch Group | 13 | -0.13 | 0.28 | 12.89 | 21.78 | 55 | | 20 | Y | N | *Metal Gear Solid Touch* | | |
|  | Super Sniper Group | 13 | -1.54 | 0.41 | 5.94 | 21.78 | 55 | | 20 | Y | N | *Super Sniper* | | |
|  | Deer Hunter Group | 13 | 0.27 | 0.28 | 12.55 | 21.78 | 55 | | 20 | Y | N | *Deer Hunter* | | |
| Okagaki & Frensch (1994) | Expt. 1, Experimental Group | 30 | 0.66 | 0.23 | 18.9 | 19.93 | 50 | | 6 | N | N | *Tetris* | | |
| Orosy-filds & Allan (1987) | Experimental Group | 10 | -1.24 | 0.42 | 5.64 | 25.4 | 80 | | 0.25 | N | N | *Centipede* | | |
| Perrot, Maillot, & Hartley (2019) | SMB Group | 11 | 0.65 | 0.33 | 9.1 | 65.55 | 64 | | 24 | Y | Y | *New Super Mario Bros* | | |
| Ruiz-Marquez, Prieto, Mayas, Toril, Reales, & Ballesteros (2019) | Active Control Group | 24 | 0.29 | 0.21 | 23.05 | 22.48 | 66 | | 7.5 | Y | Y | *The Sims* | | |
| Sanchez (2012) | "Word Whomp" Group | 30 | 0.21 | 0.18 | 29.23 | 20 | 40 | | 0.5 | Y | Y | *Word Whomp* | | |
| Schubert, Finke, Redel, Kluckow, Müller, & Strobach (2015) | Expt. 2, MoH Group | 21 | 1.03 | 0.30 | 11.41 | 24.8 | 52.38 | | 15 | Y | N | *Medal of Honor: Allied Assault* | | |
|  | Expt. 2, Tetris Group | 20 | -0.27 | 0.23 | 19.34 | 26 | 40 | | 15 | Y | Y | *Tetris* | | |
| Seçer & Satyen (2014) | Experimental group | 14 | -0.12 | 0.27 | 13.83 | 70 | 14.29 | | 3 | N | N | *Pac-Man: Adventures in Time* | | |
| Shute, Ventura, & Ke (2015) | "Video Game" Group | 42 | 0.14 | 0.16 | 41.48 | 19.7 | 57 | | 8 | N | N | *Portal 2* | | |
| Strenziok, Parasuraman, Clarke, Cisler, Thompson, & Greenwood (2014) | RON Group | 14 | -0.28 | 0.27 | 13.5 | 69.41 | 64.29 | | 36 | N | N | *Rise of Nations* | | |
| Subrahmanyam & Greenfield (1994) | Experimental Group, Females | 15 | 0.14 | 0.26 | 14.86 | 11.08 | 100 | | 2.25 | N | N | *Marble Madness* | | |
|  | Experimental Group, Males | 15 | -0.22 | 0.26 | 14.65 | 11.08 | 0 | | 2.25 | N | N | *Marble Madness* | | |
| Valdez & Ferguson (2012) | Experimental Study, 15 minute FIFAgroup | 14 | 0.83 | 0.31 | 10.35 | 21.9 | 50 | | 0.25 | Y | N | *FIFA 2010* | | |
|  | Experimental Study, 15 minute RDR-NV group | 14 | 0.45 | 0.28 | 12.82 | 19.3 | 50 | | 0.25 | Y | N | *Red Dead Redemption* | | |
|  | Experimental Study, 15 minute RDR-V group | 18 | 0.32 | 0.24 | 17.09 | 19.5 | 50 | | 0.25 | Y | N | *Red Dead Redemption* | | |
|  | Experimental Study, 45 minute FIFA group | 18 | 0.36 | 0.24 | 16.95 | 18.6 | 50 | | 0.75 | Y | N | *FIFA 2010* | | |
|  | Experimental Study, 45 minute RDR-NV group | 21 | 0.43 | 0.23 | 19.34 | 19.9 | 47.62 | | 0.75 | Y | N | *Red Dead Redemption* | | |
|  | Experimental Study, 45 minute RDR-V group | 15 | 0.39 | 0.27 | 14.01 | 21.1 | 46.67 | | 0.75 | Y | N | *Red Dead Redemption* | | |
| van Ravenzwaaij, Boekel, Fortsmann, Ratcliff, & Wagenmakers (2014) | Expt. 1, Action group | 10 | 1.04 | 0.36 | 7.83 | 20.6 | 90 | | 10 | Y | N | *Unreal Tournament 2004* | | |
|  | Expt. 1, Cognitive group | 10 | 0.24 | 0.29 | 11.68 | 20.6 | 90 | | 10 | Y | N | *The Sims 2* | | |
|  | Expt. 2, Action Group | 15 | 0.99 | 0.32 | 10.05 | 20 | 42.2 | | 10 | Y | N | *Unreal Tournament 2004* | | |
|  | Expt. 2, Cognitive Group | 15 | 0.21 | 0.26 | 14.67 | 20 | 42.2 | | 10 | Y | N | *The Sims 2* | | |
| West, Zendel, Konishi, Benady-Chorney, Bohbot, Peretz, & Belleville (2017) | Video Game Group | 8 | 2.53 | 0.73 | 1.9 | 69.3 | 50 | | 67.5 | Y | N | *Super Mario 64* | | |
| West (2020) | Strategy Game Group | 16 | 1.02 | .31 | 10.18 | 19.57 | 42 | | 9 | Y | N | *FTL: Faster Than Light* | | |
|  | Sim. Game Group | 19 | 1.56 | .34 | 8.52 | 19.57 | 42 | | 9 | Y | N | *The Sims 2* | | |
|  | Action Game Group | 18 | -1.54 | .73 | 1.9 | 19.57 | 42 | | 9 | Y | N | *Unreal Tournamet 2004* | | |
| Whitlock, McLaughlin, & Allaire (2012) | Experimental Group | 19 | 0.67 | 0.25 | 15.49 | 68.58 | 47.37 | | 14 | N | N | *World of Warcraft* | | |
| Wu & Spence (2013) | Expt. 2, Action Group | 20 | 2.92 | 0.51 | 3.79 | 21.5 | 50 | | 10 | Y | N | *Medal of Honor* | | |
|  | Expt. 2, Control Group | 20 | 0.12 | 0.22 | 19.86 | 21.5 | 50 | | 10 | Y | Y | *Ballance* | | |
|  | Expt. 2, Driving Group | 20 | 0.05 | 0.22 | 19.97 | 21.5 | 50 | | 10 | Y | N | *Need for Speed* | | |
| Wu, Cheng, Feng, D'Angelo, Alain, & Spence (2012) | Experimental Group | 16 | 1.35 | 0.35 | 5.712 | 21.3 | 56.25 | | 10 | Y | N | *Medal of Honor: Pacific Assault* | | |
|  | Control Group | 9 | 1.07 | 0.42 | 8.39 | 22 | 55.56 | | 10 | Y | Y | *Ballance* | | |

*Note.* * denotes investigations which demonstrated an overal effect size beyond two standard deviations above mean total effect size. These investigations were removed as outliers and not considered in the analyses presented.

*Note.* ** Kuhn et al., (2018) did not total hours of training, only total duration of the training intervention (six weeks)
