# Supplementary material for: A game-factors approach to cognitive benefits from video-game training: A meta-analysis
Source: PLoS One. 2023 Aug 2;18(8):e0285925. doi: 10.1371/journal.pone.0285925 (PMC10395941; doi:10.1371/journal.pone.0285925)
Supplement: S1 List — (DOCX) [file pone.0285925.s005.docx]

**S1 List. References for Included Studies**

Achtman, R. L., Green, C. S., & Bavelier, D. (2008). Video games as a tool to train visual skills. *Restorative Neurology and Neuroscience*, *26*(4–5), 435–446. https://doi.org/10.1037/0893-164X.19.4.414

Adams, D. M. (2013). *Exploring the Effect of Video Game Playing on Static and Dynamic Spatial Cognition Skills*. University of California.

Bailey, K., & West, R. (2013). The effects of an action video game on visual and affective information processing. *Brain Research, 1504*, 35–46. https://doi-org.libproxy.utdallas.edu/10.1016/j.brainres.2013.02.019

Ballesteros, S., Mayas, J., Prieto, A., Ruiz-Marquez, E., Toril, P., & Reales, J. M. (2017). Effects of video game training on measures of selective attention and working memory in older adults: Results from a randomized controlled trial. *Frontiers in Aging Neuroscience, 9.* https://doi-org.libproxy.utdallas.edu/10.3389/fnagi.2017.00354

Basak, C., Boot, W. R., Voss, M. W., & Kramer, A. F. (2008). Can training in a real-time strategy video game attenuate cognitive decline in older adults? *Psychology and Aging*, *23*(4), 765–777. https://doi.org/10.1037/a0013494

Bejjanki, V. R., Zhang, R., Li, R., Pouget, A., Green, C. S., Lu, Z.-L., & Bavelier, D. (2014). Action video game play facilitates the development of better perceptual templates. *Proceedings of the National Academy of Sciences*, *111*(47), 16961–16966. https://doi.org/10.1073/pnas.1417056111

Belchior, P., Marsiske, M., Sisco, S. M., Yam, A., Bavelier, D., Ball, K., & Mann, W. C. (2013). Video game training to improve selective visual attention in older adults. *Computers in Human Behavior*, *29*(4), 1318–1324. https://doi.org/10.1016/j.chb.2013.01.034

Belchior, P., Yam, A., Thomas, K. R., Bavelier, D., Ball, K. K., Mann, W. C., & Marsiske, M. (2019). Computer and Videogame Interventions for Older Adults’ Cognitive and Everyday *Functioning. Games For Health Journal*, 8(2), 129–143. https://doi-org.libproxy.utdallas.edu/10.1089/g4h.2017.0092

Blacker, K. J., Curby, K. M., Klobusicky, E., & Chein, J. M. (2014). Effects of Action Video Game Training on Visual Working Memory. *Journal of Experimental Psychology. Human Perception and Performance*, *40*(5), 1992–2004. https://doi.org/10.1037/a0037556

Boot, W. R., Champion, M., Blakely, D. P., Wright, T., Souders, D. J., & Charness, N. (2013). Video games as a means to reduce age-related cognitive decline: attitudes, compliance, and effectiveness. *Frontiers in Psychology*, *4*(February), 31. https://doi.org/10.3389/fpsyg.2013.00031

Boot, W. R., Kramer, A. F., Simons, D. J., Fabiani, M., & Gratton, G. (2008). The effects of video game playing on attention, memory, and executive control. *Acta Psychologica*, *129*(3), 387–398. https://doi.org/10.1016/j.actpsy.2008.09.005

Cherney, I. D., Bersted, K., & Smetter, J. (2014). Training Spatial Skills in Men and Women. *Perceptual and Motor Skills*, *119*(1), 82–99. https://doi.org/10.2466/23.25.PMS.119c12z0

Chiappe, D., Conger, M., Liao, J., Caldwell, J. L., & Vu, K. P. L. (2013). Improving multi-tasking ability through action videogames. *Applied Ergonomics*, *44*(2), 278–284. https://doi.org/10.1016/j.apergo.2012.08.002

Clark, J. E., Lanphear, A. K., & Riddick, C. C. (1987). The effects of video game playing on the response selection of elderly adults. *Journal of Gerontology*, *42*(1), 82–85.

Clemenson, G. D., & Stark, C. E. L. (2015). Virtual Environmental Enrichment through Video Games Improves Hippocampal-Associated Memory. *Journal of Neuroscience*, *35*(49), 16116–16125. https://doi.org/10.1523/JNEUROSCI.2580-15.2015

Cohen, J. E., Green, C. S., & Bavelier, D. (2008). Training visual attention with video games: Not all games are created equal. *Computer Games and Team and Individual Learning*.

Colzato, L. S., van den Wildenberg, W. P. M., & Hommel, B. (2019). Cognitive control and the COMT Val158Met polymorphism: Genetic modulation of videogame training and transfer to task -switching efficiency. *ESCOP 2013: 18th Meeting of the European Society for Cognitive Psychology, Budapest, Hungary, August 29-September 1, 2013 [Abstracts]*, 88. https://doi-org.libproxy.utdallas.edu/10.1037/e636952013-110

De Lisi, R, & Wolford, J. L. (2002). Improving children’s mental rotation accuracy with computer game playing. *Journal of Genetic Psychology*, *163*(3), 272–282. https://doi.org/10.1080/00221320209598683

De Lisi, R. & Cammarano, D. M. (1996). Computer experience and gender differences in undergraduate mental rotation performance. *Computers in Human Behavior*, *12*(3), 351–361. https://doi.org/10.1016/0747-5632(96)00013-1

Dorval, M., & Pépin, M. (1986). Effect of Playing a Video Game on a Measure of Spatial Visualization. *Perceptual Motor Skills*, *62*, 159–162.

Feng, J., Spence, I., & Pratt, J. (2007). Playing an action video game reduces gender differences in spatial cognition. *Psychological Science : A Journal of the American Psychological Society / APS*, *18*(10), 850–855. https://doi.org/10.1111/j.1467-9280.2007.01990.x

Gagnon, D. (1986). *Interactive versus observational media: the influence of user control and cognitive styes on spatial learning* (unpublished doctoral dissertaition), Harvard University, Cambridge, Massachusetts.

Glass, B. D., Maddox, W. T., & Love, B. C. (2013). Real-Time Strategy Game Training: Emergence of a Cognitive Flexibility Trait. *PLoS ONE*, *8*(8), 1–8. https://doi.org/10.1371/journal.pone.0070350

Goldstein, J., Cajko, L., Oosterbroek, M., Michielsen, M., van Houten, O., & Saverda, F. (1997). Video Games and the Elderly. *Social Behavior and Personality*, *25*(4), 345–352. Retrieved from http://www.savie.qc.ca/BaseConnaissances/upload/pdf/3029_goldsteinetal_1997_videogamesandtheelderly_Index3029.pdf

Gonzales, C. J. (2012). How expertise can modulate spatial attention within and across sensory modalities: The case of video game players. *Dissertation Abstracts International: Section B: The Sciences and Engineering*. https://doi.org/10.1007/978-90-481-9449-0

Green, C. S., & Bavelier, D. (2006b). Effect of action video games on the spatial distribution of visuospatial attention. *Journal of Experimental Psychology. Human Perception and Performance*, *32*(6), 1465–1478. https://doi.org/10.1037/0096-1523.32.6.1465

Green, C. S., Li, R., & Bavelier, D. (2010). Perceptual learning during action video game playing. *Topics in Cognitive Science*, *2*(2), 202–216. https://doi.org/10.1111/j.1756-8765.2009.01054.x

Green, C. S., Sugarman, M. a., Medford, K., Klobusicky, E., & Bavelier, D. (2012). The effect of action video game experience on task-switching. *Computers in Human Behavior*, *28*(3), 984–994. https://doi.org/10.1016/j.chb.2011.12.020

Huang, K.T. (2020). Exergaming Executive Functions: An Immersive Virtual Reality-Based Cognitive Training for Adults Aged 50 and Older. *Cyberpsychology, Behavior and Social Networking*, 23(3), 143–149. https://doi-org.libproxy.utdallas.edu/10.1089/cyber.2019.0269

Hutchinson, C. V, Barrett, D. J. K., Nitka, A., & Raynes, K. (2015). Action video game training reduces the Simon Effect. *Psychonomic Bulletin & Review*, 587–592. https://doi.org/10.3758/s13423-015-0912-6

Kühn, S., Gleich, T., Lorenz, R. C., Lindenberger, U., & Gallinat, J. (2014). Playing Super Mario induces structural brain plasticity: gray matter changes resulting from training with a commercial video game. *Molecular Psychiatry*, *19*(2), 265–271. https://doi.org/10.1038/mp.2013.120

Kühn, S., Berna, F., Lüdtke, T., Gallinat, J., & Moritz, S. (2018). Fighting depression: Action video game play may reduce rumination and increase subjective and objective cognition in depressed patients. *Frontiers in Psychology*, 9. https://doi-org.libproxy.utdallas.edu/10.3389/fpsyg.2018.00129

Li, L., Chen, R., & Chen, J. (2016). Playing Action Video Games Improves Visuomotor Control. *Psychological Science*, *27*(8), 1092–1108. https://doi.org/10.1177/0956797616650300

Li, R., Polat, U., Scalzo, F., & Bavelier, D. (2010). Reducing backward masking through action game training. *Journal of Vision*, *10*(14), 33–33. https://doi.org/10.1167/10.14.33

Martincevic, M. & Vranic, A. (2020). Casual Game or Cognitive Gain: Multitask Casual Game as a Training for Young Adults. *Journal of Cogntive Enhancement*. https://doi.org/10.1007/s41465-020-00173-5

McCord, A., Cocks, B., Barreiros, A. R., & Bizo, L. A. (2020). Short video game play improves executive function in the oldest old living in residential care. *Computers in Human Behavior,* 108. https://doi-org.libproxy.utdallas.edu/10.1016/j.chb.2020.106337

McDermott, A. F. (2013). A comparison of two video game genres as cognitive training tools in older adults. *ProQuest Dissertations and Theses*. Retrieved from http://search.proquest.com/docview/1362257897?accountid=10906%5Cnhttp://zsfx.lib.iastate.edu:3410/sfxlcl41?url_ver=Z39.88-2004&rft_val_fmt=info:ofi/fmt:kev:mtx:dissertation&genre=dissertations+%26+theses&sid

Minear, M., Brasher, F., Guerrero, C. B., Brasher, M., Moore, A., & Sukeena, J. (2016). A simultaneous examination of two forms of working memory training: Evidence for near transfer only. *Memory and Cognition*, *44*(7), 1014–1037. https://doi.org/10.3758/s13421-016-0616-9

Momi, D., Smeralda, C., Sprugnoli, G., Neri, F., Rossi, S., Rossi, A., … Santarnecchi, E. (2019). Thalamic morphometric changes induced by first‐person action videogame training. *European Journal of Neuroscience*, 49(9), 1180–1195. Retrieved from https://search-ebscohost-com.libproxy.utdallas.edu/login.aspx?direct=true&db=psyh&AN=2019-32996-007&site=ehost-live

Nelson, R. A., & Strachan, I. (2009). Action and puzzle video games prime different speed/accuracy tradeoffs. *Perception*, *38*(11), 1678–1687. https://doi.org/10.1068/p6324

Nouchi, R., Taki, Y., Takeuchi, H., Hashizume, H., Akitsuki, Y., Shigemune, Y., … Kawashima, R. (2012). Brain training game improves executive functions and processing speed in the elderly: A randomized controlled trial. *PLoS ONE*, *7*(1). https://doi.org/10.1371/journal.pone.0029676

Nouchi, R., Kawata, N. Y. D. S., Saito, T., Himmelmeier, R. M., Nakamura, R., Nouchi, H., & Kawashima, R. (2020). Dorsolateral Prefrontal Cortex Activity during a Brain Training Game Predicts Cognitive Improvements after Four Weeks’ Brain Training Game Intervention: Evidence from a Randomized Controlled Trial. *Brain Sciences, 10*(8). https://doi-org.libproxy.utdallas.edu/10.3390/brainsci10080560

Novak, E., & Tassell, J. (2015). Using video game play to improve education-majors’ mathematical performance: An experimental study. *Computers in Human Behavior*, *53*, 124–130. https://doi.org/10.1016/j.chb.2015.07.001

Oei, A. C., & Patterson, M. D. (2013). Enhancing Cognition with Video Games: A Multiple Game Training Study. *PLoS ONE*, *8*(3). https://doi.org/10.1371/journal.pone.0058546

Oei, A. C., & Patterson, M. D. (2014a). Playing a puzzle video game with changing requirements improves executive functions. *Computers in Human Behavior*, *37*, 216–228. https://doi.org/10.1016/j.chb.2014.04.046

Oei, A. C., & Patterson, M. D. (2015). Enhancing perceptual and attentional skills requires common demands between the action video games and transfer tasks*. Frontiers in Psychology,* 6. Retrieved from https://search-ebscohost-com.libproxy.utdallas.edu/login.aspx?direct=true&db=psyh&AN=2016-24071-001&site=ehost-live

Okagaki, L., & Frensch, P. A. (1994). Effects of video game playing on measures of spatial performance: Gender effects in late adolescence. *Journal of Applied Developmental Psychology*, *15*(1), 33–58. https://doi.org/10.1016/0193-3973(94)90005-1

Orosy-fildes, C., & Allan, R. W. (1987). Psychology of computer use: XII. Videogame Play: Human Reaction Time to Visual Stimuli. *Peceptual and Motor Skills*, *69*, 243–247.

Perrot, A., Maillot, P., & Hartley, A. (2019). Cognitive Training Game Versus Action Videogame: Effects on Cognitive Functions in Older Adults. *Games For Health Journal,* 8(1), 35–40. https://doi-org.libproxy.utdallas.edu/10.1089/g4h.2018.0010

Ruiz-Marquez, E., Prieto, A., Mayas, J., Toril, P., Reales, J. M., & Ballesteros, S. (2019). Effects of Nonaction Videogames on Attention and Memory in Young Adults. *Games For Health Journal.* https://doi-org.libproxy.utdallas.edu/10.1089/g4h.2019.0004

Sanchez, C. A. (2012). Enhancing visuospatial performance through video game training to increase learning in visuospatial science domains. *Psychonomic Bulletin and Review*, *19*(1), 58–65. https://doi.org/10.3758/s13423-011-0177-7

Schubert, T., Finke, K., Redel, P., Kluckow, S., Müller, H., & Strobach, T. (2015). Video game experience and its influence on visual attention parameters: An investigation using the framework of the Theory of Visual Attention (TVA). *Acta Psychologica*, *157*, 200–214. https://doi.org/10.1016/j.actpsy.2015.03.005

Seçer, I., & Satyen, L. (2014). Video Game Training and Reaction Time Skills Among Older Adults. *Activities, Adaptation and Aging*, *38*(3), 220–236. https://doi.org/10.1080/01924788.2014.935908

Shute, V. J., Ventura, M., & Ke, F. (2015). The power of play: The effects of Portal 2 and Lumosity on cognitive and noncognitive skills. *Computers and Education*, *80*, 58–67. https://doi.org/10.1016/j.compedu.2014.08.013

Strenziok, M., Parasuraman, R., Clarke, E., Cisler, D. S., Thompson, J. C., & Greenwood, P. M. (2014). Neurocognitive enhancement in older adults: Comparison of three cognitive training tasks to test a hypothesis of training transfer in brain connectivity. *NeuroImage*, *85*, 1027–1039. https://doi.org/10.1016/j.neuroimage.2013.07.069

Subrahmanyam, K., & Greenfield, P. M. (1994). Effect of video game practice on spatial skills in girls and boys. *Journal of Applied Developmental Psychology*, *15*(1), 13–32. https://doi.org/10.1016/0193-3973(94)90004-3

Valadez, J. J., & Ferguson, C. J. (2012). Just a game after all: Violent video game exposure and time spent playing effects on hostile feelings, depression, and visuospatial cognition. *Computers in Human Behavior*, *28*(2), 608–616. https://doi.org/10.1016/j.chb.2011.11.006

van Ravenzwaaij, D., Boekel, W., Forstmann, B. U., Ratcliff, R., & Wagenmakers, E.-J. (2014). Action Video Games Do Not Improve the Speed of Information Processing in Simple Perceptual Tasks. *Journal of Experimental Psycholog: General*, *143*(5), 1794–1805. https://doi.org/10.1037/a0036923.Action

West, G. L., Zendel, B. R., Konishi, K., Benady-Chorney, J., Bohbot, V. D., Peretz, I., & Belleville, S. (2017). Playing Super Mario 64 increases hippocampal grey matter in older adults. *PLoS ONE,* 12(12). https://doi-org.libproxy.utdallas.edu/10.1371/journal.pone.0187779

West, R., Swing, E. L., Anderson, C. A., & Prot, S. (2020). The Contrasting Effects of an Action Video Game on Visuo-Spatial Processing and Proactive Cognitive Control. *International Journal of Environmental Research and Public Health, 17*(14). https://doi-org.libproxy.utdallas.edu/10.3390/ijerph17145160

Whitlock, L. A., McLaughlin, A. C., & Allaire, J. C. (2012). Individual differences in response to cognitive training: Using a multi-modal, attentionally demanding game-based intervention for older adults. *Computers in Human Behavior*, *28*(4), 1091–1096. https://doi.org/10.1016/j.chb.2012.01.012

Wu, S., Cheng, C.K., Feng, J., D’Angelo, L., Alain, C., & Spence, I. (2012). Playing a First-person Shooter Video Game Induces Neuroplastic Change. Journal of Cognitive *Neuroscience, 24*(6), 1286–1293. https://doi-org.libproxy.utdallas.edu/10.1162/jocn_a_00192

Wu, S., & Spence, I. (2013). Playing shooter and driving videogames improves top-down guidance in visual search. *Attention, Perception, & Psychophysics*, *75*(4), 673–686. https://doi.org/10.3758/s13414-013-0440-2
