# Supplementary material for: A game-factors approach to cognitive benefits from video-game training: A meta-analysis
Source: PLoS One. 2023 Aug 2;18(8):e0285925. doi: 10.1371/journal.pone.0285925 (PMC10395941; doi:10.1371/journal.pone.0285925)
Supplement: Attachment — Submitted filename: REVIEW OF PONE-D-22-06647.docx [file pone.0285925.s007.docx]

REVIEW OF PONE-D-22-06647

A Game-Factors Approach to Cognitive Benefits from Video-Game training: A Meta- Analysis

The study provides an interesting meta-analytic approach to study the impact of video game training on cognition, focusing on game features that are thought to characterize distinct video game genres. This is an original approach that will make an interesting contribution to the field and hopefully should stimulate further research. I have some clarification questions and also some suggestions that aim at strengthening some results and conclusions.

The present meta-analysis was pre-registered on OSF and I had access to the PRISMA checklist but not to the full methods. Therefore, the comments below are based on the information provided in the manuscript only, without taking the pre-registered methods analysis plan and hypotheses into consideration.

My primary concern relates to the use of within-subject effect sizes, which do not properly control for numerous confounds such as practice or test-retest effects or placebo effects. While I understood that this was necessary to include control games, the use of effect sizes based on pre-post change scores has important limitations that should be acknowledged [(Cuijpers et al., 2017)](https://www.zotero.org/google-docs/?53Opq7). My second and third main concerns are related to the analytic strategy (assuming independent effect sizes, using r = 0.5) and the lack of details regarding the criteria used for coding game features, genres, formats, and cognitive constructs. I felt that the paper would benefit from clarifying both the choice of categories (genre vs format, experimental vs control) and the levels (action vs strategy, serious vs. casual).

*However, given that the study was pre-registered,* I wasn’t able to determine if there were deviations from the registered methods (coding), analysis plan (effect sizes and models) and hypotheses tested (number of analyses). If an analysis appears sub-optimal but was registered, any change or any additional analysis, even if it represents an improvement, should be strongly justified and yet considered exploratory as it will deviate from the registered analysis plan.

Below I provide more details about each specific point. I am confident that the authors can address most of them in a reply, and that providing some of the additional analyses suggested in this review will strengthen their results and thereby increase the impact of their study.

1. **Effect sizes from within-subject designs pose several problems.**

***Confounds.*** The first relates to the interpretation and presence of confounds. As is now standard in intervention studies, the use of a control group is essential to rule out potential confounds, such as placebo, test-retest or practice effects. This is also true in meta-analyses that aim to compare different groups or interventions.

***Between group comparisons.*** Second, the use of within-subject effects also makes between-group comparisons less reliable as the Q statistics controls for only one source of heterogeneity related to within-group variance and not for the variance of between-group differences in pre-test performance, post-test performance or change scores, that are essential for comparing subgroups.

***Pre-post correlation.*** A third problem arises from the correlation between pre-test and post-test which is almost never reported in primary studies and thus calls for adjustments of the effect sizes and their variance. While assuming a prepost correlation of r = 0.5 is a neutral (i.e., unbiased) choice, this choice will impact the estimates, their variance (hence the weights) and thereby the results. It would be reassuring to see sensitivity analyses testing the impact of different values of r and this would certainly strengthen the results and conclusions. This is particularly important given that the meta-analysis is based on within-subject change scores and the correlation between pretest and postest can thus be expected to be higher than 0.5. Yet, sensitivity analyses testing several values below and above 0.5 would seem advisable.

2. **The analysis strategy raises several questions.**

Several analytical choices are questionable (potentially suboptimal) and thus need better justification.

***Random effects model.*** The authors use random effects models, which assume dependent effect sizes. Yet, the shared excel files (which are hardly readable and would be much more useful as downloadable excel files) suggest that the authors extracted more than one effect size from each study and group of participants. Were effect sizes coming from the same study aggregated or randomly selected and how? If averaged, how was variance handled? If randomly selected, did the author assess sensitivity via bootstrapping? Because effect sizes from the same study may not be fully independent (even if coming from different groups), an alternative may be to use methods that can handle dependent effect sizes, such as multilevel methods or meta-analytic models using robust variance estimates which allow to specify a correlated or hierarchical structure of the weights.

***Moderators*** were assessed across multiple subgroup analyses. Were all analyses pre-registered and if yes did the authors consider correcting for multiple testing? One drawback of this approach is that it assesses the impact of each moderator individually, without controlling for other moderators, some of which may covary (e.g., genre, format, features, participants age). Meta-regression models can assess the influence of several moderators simultaneously. Meta-regression was used to assess continuous moderators and a similar approach may be recommended at least for some analyses of categorical (or dummy coded) moderators. Finally, some subgroup analyses may be underpowered as they rely on relatively small numbers of studies or effects.

***Publication bias*** analyses are limited to trim and fill, which has been extensively criticized. There are a multitude of methods available now, each with its own pros and cons, that are generally recommended over the trim and fill approach. An increasingly common approach is to run publication bias sensitivity analyses using a variety of techniques [(Carter et al., 2019; Mathur & VanderWeele, 2020)](https://www.zotero.org/google-docs/?tL0KYM). Moregenerall, I highly recommend these two recently published meta-analyses which provide state-of-the-art methods in terms of meta-analytic models for dependent effect sizes, moderator analysis and publication bias detection [(Coles et al., 2019; Lehtonen et al., 2018)](https://www.zotero.org/google-docs/?uWOWFI).

**3. The coding raises numerous questions and could potentially be improved.**

The introduction and discussion largely argue that the existing literature has emphasized an association between video game features and their cognitive effects and the present study attempts to address this hypothesis. Yet, I believe that the coding could be improved to better reflect the authors’ hypotheses as coding was sometimes inconsistent with the authors argument. Some games could be arguably coded in a different genre and some tasks in different constructs which might change the results and conclusions.

***Coding of game features.*** The part I liked most concerns the coding of game features which I found particularly interesting and could have been pushed even further. For example, a theory-grounded coding mapping specific game mechanics with particular cognitive demands (rather than mechanics associated with genres) would probably allow a different set of testable predictions.

***Coding of game genre and format.*** What criteria were used to define genres and format? casual and serious games have been considered as video game genres in other studies, suggesting these 2 categories may overlap at least partially. Aren’t action, strategy, casual and serious meant to be mutually exclusive categories? How was the game Tetris (from Belchior et al) classified? It appears in both action and casual — which would seem wrong to me? How about Portal 2? Was it considered an action game and on which criteria? Were serious games only commercial games (how about cognitive training like cogmed, lumosity)? How about brain training games? Are these considered casual or serious? These notions should be clarified. Note that some codes (e.g. format, serious) were missing from the shared word doc and that the excel files were hardly readable or usable (no possibility to search). Also, the word “commercial games” is mentioned for the first time in the discussion line 453. Does that refer to off-the-shelf games? Was that an inclusion criterion? Were all serious games commercial games?

There have been debates and inconsistencies in how genre was defined and handled in prior meta-analyses (either as selection/inclusion criteria or as moderator) and I am not clear if and how format differs from genre. This is a central point of the manuscripts that deserved some clarification including whether and why these codes are considered independent.

Also, the genres Action and Strategy may be (arguably) too restrictive to capture all games. Forcing all games to fit into these 2 genres results in categories that are too heterogeneous to be meaningful. For example, the Action genre may not be the best fit for games like Centipede, Donkey Kong, Pacman or Wii Fit… Similarly, games like Angry Birds, The Sims or Tetris do not seem to fit well into the Strategy genre. Others have considered categories like simulation, exergame or platform games or even brain training. The literature search mentions brain training games but I didn’t see any. Were brain training games excluded and why? And if not how were these games coded?

More importantly, the categorization of games into genres seems inconsistent with the authors' argument that games that belong to a given genre share features that are responsible for certain cognitive effects.

***Coding of experimental and control games.***

Here too, the reader would benefit from a more detailed description of how this coding was performed as it wasn’t available in the supplementary excel file. Note that the coding of serious games is also missing… Again, these are important as the criteria for defining serious games or experimental vs control games has varied across studies.

***Coding of cognitive constructs.***

Relatedly, a similar reflection would also be relevant for defining cognitive constructs of interest. What criteria guided the choice of these 3 outcome categories: attention/perception, higher cognition and psychosocial? Previous meta-analyses of video games (and in other fields too) have categorized cognitive measures into separable constructs may provide some guidance or inspiration (e.g. [Bediou et al., 2018; Powers et al., 2013; Powers & Brooks, 2014; Sala et al., 2018; Wang et al., 2017)](https://www.zotero.org/google-docs/?i9pu3M). What is the rationale for including some memory (working memory) tasks in higher cognition, whereas others are coded as memory? Psychosocial also includes depression and anxiety scales, affect or emotion (PANAS), risk taking… which may not really correspond to psychosocial skills. Again, the categories appear too heterogeneous to be meaningful. For example, memory may include tasks that include manipulation of verbal or visuo spatial material which may be differentially impacted by distinct games. Studies of memory may also include older participants and although the effect is NS, the trend is negative suggesting smaller effects in older participants (consistent with previous meta-analyses that treated age as categorical that report smaller effects in older adults).

**4. Discussion: relationship between features, genres, format, etc.**

The authors conclude that game features predict differential effects on cognition. Yet, their effect sizes do not differentiate between skills as the confidence intervals overlap and the use of within-subject effects does not allow proper between-group comparisons. Therefore, some conclusions may need to be toned down or rephrased and these limitations should be better acknowledged.

***Mapping between features and cognitive constructs.*** The game features coded do not clearly map onto specific cognitive demands, which appears to contradict what the authors discuss about the choice of experimental and control groups in primary studies.

Lines 465-477, *“Importantly, “control” condition games failed to produce any significant transfer to any cognitive construct, despite a diverse range of games being presented as active control conditions (see Supplementary Table 1). This is evidence that, in general, VG-based interventions have tailored their cognitive transfer measures specifically to be sensitive to the cognitive demands of the intervention, and/or have intentionally selected control games not expected to produce transfer to the constructs examined. While this is an understandable design strategy, this practice may lead authors to incorrectly conclude that the control games used, or aspects thereof, do not produce cognitive improvements. As a concrete example of why this is problematic, consider the games Tetris and The Sims. Both games have been frequently used as active control conditions in studies of “action” video games, and producing negligible transfer as expected [12, 13, 37]. However, both of those games have demonstrated significant transfer, in studies in which they were an intervention of interest.”*

The cases of Tetris and The Sims are particularly illustrative and interesting as the choice of using these as experimental or control games may depend on which aspect of cognition was measured (and considered as demonstrating transfer). This seems to contradict another statement that appears a few lines later. Lines 500-510: *“the above definition of “action” games would translate to games that are combat-focused with time pressure, and have an active opponent. However, the current meta-analysis found consistent transfer to measures of attention and perception was facilitated by games that featured a first-person perspective, a single controllable object, a single win state, lacked an active opponent, and were primarily non-combat games. Not only was transfer to the attention and perception construct found to be agnostic to the presence of time pressure, but both active opponents and combat gameplay explicitly failed to produce consistent transfer.* ***This finding provides evidence that the post-hoc justifications given in the past explaining the link between “action” video game play and enhanced attention and perception may be inaccurate.”***

These are strong statements and conclusions which depend largely on the appropriate coding of game features, video game genres and cognitive construct, as well as their appropriate analysis allowing more direct comparisons which were not possible here.

Finally, the sentence line 566-567: *“The gameplay factors analysis conducted in this meta-analysis rebuffs the “action” vs “strategy” distinction as useful.”* appears too strong and should be downplayed given the results and the rest of the discussion arguing that this distinction has some value. This study **sheds some** light on how some of the game features that are present in action and/or strategy games and may be associated with their (differential?) cognitive effects. However, I don’t think it ***rebuffs*** the distinction between action and strategy games… As the authors rightfully discuss on line 573, the “*genre distinction does have merit”* (as well as some “historical” value) as action and strategy games were initially quite distinct in terms of mechanics, features and cognitive demands, and thus represented meaningful categories less than 10 years ago. However, as the video game ecosystem grows and new genres, hybrid genres and mixed genres arise, the features that were once specific to action or strategy games have become more and more common across genres and thus more difficult to isolate (see [(Dale et al., 2020; Dale & Green, 2017)](https://www.zotero.org/google-docs/?DGHT4u). Toning down the conclusion won’t minimize the potential impact of this study which will make an important contribution to the field and should stimulate more work looking at how game characteristics map onto cognitive demands and whether they predict cognitive effects.

The authors argue that the lack of effect in the control condition *“is evidence that, in general, VG-based interventions have tailored their cognitive transfer measures specifically to be sensitive to the cognitive demands of the intervention.”* (line 468). This conclusion would require an analysis based on the cognitive demands rather than the game characteristics –which were not coded here– unless there is a perfect match between game features (e.g. pace or time pressure) and cognitive demands (attentional control)?

**5. Additional (minor) comments.**

***Study quality / risk of bias.*** Details about the PEDro scale would be appreciated, especially because it is less commonly used than other tools such as the Cochrane risk of bias scale. (<https://guides.himmelfarb.gwu.edu/systematic_review/reporting-quality-risk-of-bias>).

***Participant and study characteristics.***

Meta-regressions show non-significant modulation by either participant or study characteristics. Were these moderating influences also considered in subgroup analyses? Some factors tend to covary (e.g., sample size, age, training duration) and may thus be difficult to disentangle.

Participants characteristics:

Are groups matched in gender? Proportion (%) females overall can be misleading if the groups are not balanced.

Why was average age treated as continuous… previous work has mostly used age-group categories (e.g., children, young adults, old adults).

Study characteristics:

Number of participants is already included in the weights (inverse variance) and therefore I don’t think it needs to be added to the models.

What was the rationale for coding the number of cognitive outcomes? What result was expected?

It is surprising that total hours had no effect but again there may be complex interactions with participant characteristics, cognitive construct, game features or genres.

**6. Citations**

[Bediou, B., Adams, D. M., Mayer, R. E., Tipton, E., Green, C. S., & Bavelier, D. (2018). Meta-analysis of action video game impact on perceptual, attentional, and cognitive skills. *Psychological Bulletin*, *144*(1), 77–110. https://doi.org/10.1037/bul0000130](https://www.zotero.org/google-docs/?htX1HF)

[Carter, E. C., Schönbrodt, F. D., Gervais, W. M., & Hilgard, J. (2019). Correcting for Bias in Psychology: A Comparison of Meta-Analytic Methods. *Advances in Methods and Practices in Psychological Science*, *2*(2), 115–144. https://doi.org/10.1177/2515245919847196](https://www.zotero.org/google-docs/?htX1HF)

[Coles, N. A., Larsen, J. T., & Lench, H. C. (2019). A meta-analysis of the facial feedback literature: Effects of facial feedback on emotional experience are small and variable. *Psychological Bulletin*, *145*(6), 610–651. https://doi.org/10.1037/bul0000194](https://www.zotero.org/google-docs/?htX1HF)

[Cuijpers, P., Weitz, E., Cristea, I. A., & Twisk, J. (2017). Pre-post effect sizes should be avoided in meta-analyses. *Epidemiology and Psychiatric Sciences*, *26*(4), 364–368. https://doi.org/10.1017/S2045796016000809](https://www.zotero.org/google-docs/?htX1HF)

[Dale, G., & Green, C. S. (2017). The Changing Face of Video Games and Video Gamers: Future Directions in the Scientific Study of Video Game Play and Cognitive Performance. *The Journal of Cognitive Enhancement*, *in press*.](https://www.zotero.org/google-docs/?htX1HF)

[Dale, G., Joessel, A., Bavelier, D., & Green, C. S. (2020). A new look at the cognitive neuroscience of video game play. *Annals of the New York Academy of Sciences*, nyas.14295. https://doi.org/10.1111/nyas.14295](https://www.zotero.org/google-docs/?htX1HF)

[Lehtonen, M., Soveri, A., Laine, A., Järvenpää, J., de Bruin, A., & Antfolk, J. (2018). Is bilingualism associated with enhanced executive functioning in adults? A meta-analytic review. *Psychological Bulletin*, *144*(4), 394–425. https://doi.org/10.1037/bul0000142](https://www.zotero.org/google-docs/?htX1HF)

[Mathur, M. B., & VanderWeele, T. J. (2020). Sensitivity analysis for publication bias in meta-analyses. *Journal of the Royal Statistical Society. Series C, Applied Statistics*, *69*(5), 1091–1119. https://doi.org/10.1111/rssc.12440](https://www.zotero.org/google-docs/?htX1HF)

[Powers, K. L., & Brooks, P. J. (2014). Evaluating the Specificity of Effects of Video Game Training. In Fran Blumberg (Ed.), *Learning by playing: Frontiers of video gaming in education,* (pp. 302–329). Oxford University Press.](https://www.zotero.org/google-docs/?htX1HF)

[Powers, K. L., Brooks, P. J., Aldrich, N. J., Palladino, M. A., & Alfieri, L. (2013). Effects of video-game play on information processing: A meta-analytic investigation. *Psychon Bull Rev*, *20*(6), 1055–1079. https://doi.org/10.3758/s13423-013-0418-z](https://www.zotero.org/google-docs/?htX1HF)

[Sala, G., Tatlidil, K. S., & Gobet, F. (2018). Video game training does not enhance cognitive ability: A comprehensive meta-analytic investigation. *Psychological Bulletin*, *144*(2), 111–139. https://doi.org/10.1037/bul0000139](https://www.zotero.org/google-docs/?htX1HF)

[Wang, P., Liu, H. H., Zhu, X. T., Meng, T., Li, H. J., & Zuo, X. N. (2017). Action Video Game Training for Healthy Adults: A Meta-Analytic Study. *Front Psychol*, *7*. https://doi.org/doi: 10.3389/fpsyg.2016.00907](https://www.zotero.org/google-docs/?htX1HF)
