# Supplementary material for: A game-factors approach to cognitive benefits from video-game training: A meta-analysis
Source: PLoS One. 2023 Aug 2;18(8):e0285925. doi: 10.1371/journal.pone.0285925 (PMC10395941; doi:10.1371/journal.pone.0285925)
Supplement: Attachment — Submitted filename: Smith & Basak 2022 Response to ReviewersFinal.docx [file pone.0285925.s008.docx]

**Response to Editor and the Reviewers**

We sincerely thank the reviewers and the Editor for their insightful comments and we have addressed all of these comments in the revised manuscript that has undergone substantial changes.

Please find our specific responses below, where all comments are in Italics and our responses are in regular font:

**Editor Comments**

1. *First, Similarly to Reviewer 2, I thought that the evaluation of the paper was compromised by some accessibility issues regarding the preregistration and the data. With regards to the preregistration, the project is private and hence we could not read the preregistration. The text also does not indicate which hypotheses were preregistered neither which analyses are part of the preregistration and which ones were exploratory. The text should be revised to clearly identify these. With regards to the data, it seemed to be some sort of image of an excel file, which unfortunately is not useful for sharing data since one cannot reuse the data (unless one retypes all of the information visually available). I wanted to explore the data-set, but one can neither download it, see it closely, click on it or anything. Please be aware that sharing of data should follow the FAIR principles (findable, accessible, interoperable, and reusable). These two points will be certainly critical in subsequent evaluation of your paper, if you decide to submit a revision.*
   a. The accessibility issues expressed by the editors and reviewers were not intended by the authors – we are fully committed to providing access to the dataset utilized in this study. The difficulties in access the shared dataset appear to stem from accessibility restrictions enforced by the authors’ home university, which we believe we have addressed. The shared materials should now be fully viewable and editable by any party with an access link – please contact the corresponding author (Chandramallika Basak) if this is not the case, as further access issues are erroneous.
2. *Second, it stroke me that most the of the factors included in the analyses of the games that were not significant, still generated effects that went in the same direction as the significant ones, often with similar mean size (but sometimes even larger) although lacking significance. Given that these cases were also often the cases with less studies (small k), it seems to me that the difference between significant and non-significant is probably not very informative. This should be taken in consideration. Can one be certain that one factor is more conducive than the other in generating a transfer effect? Please consider this issue carefully. One possible approach to mitigate this issue would be to perform a Bayesian Meta-analysis and derive uncertainty estimates. Alternatively, you should provide the reader with some sort of evidence of which differences observed with the current analysis are meaningful, and provide the caveats explaining the ones that are yet not credible (for example, due to the lack of sufficient studies included).*
   a. After carefully considering this comment (as well as those from reviewer 2 who expressed similar misgivings re. our statistical approach), we decided to re-analyze our data using a mixed-effect meta-regression approach – see lines 253-257 of the revised method section, as well as the revised publication bias analysis (lines 301-310). This approach allows us to a) compare and correct for all of our variable of interest in a single model, b) reduces the total number of comparisons (a consequence of point a), c) directly compare the effect of subgroups on our outcome measures, and d) incorporate a correction for publication bias (via Egger’s regression test) into the models natively. We feel this revised approach addresses all of the major concerns provided by the reviewers and the editor, and is a much stronger statistical approach overall.

_____________________________________________________________________________________

**Reviewer 1 Comments:**

1. *why is the physiotherapy evidence database chosen and were studies excluded based on this assessment?*
   a. More information regarding the PEDro scale as well as justification for its use have been added in lines 134-138
   b. This was not an exclusion criteria, but was examined as a factor of study quality.
2. *why are age and percentage female chosen as moderators as well as publication characteristics – I think that overall moderator choice deserve a better motivation*
   a. they are not – average age and percentage female are *participant characteristics*, distinct from *publication characteristics*. However, this distinction is no longer relevant in the revised statistical approach taken in the re-submitted manuscript – participant characteristics, publication quality characteristics, and all of the binary factors considered in the initial analysis (genre, format, study group, game factors) have been modeled as fixed effects in a mixed-effect meta-regression model (see lines 253-60 and lines 324-338)
3. *to the gameplay factors: again, I miss a somewhat motivated choice why those factors ( Movement Style (egocentric vs. allocentric method of spatial navigation), Perspective (1st person vs 3rd person viewing perspective), number of Controllable Objects (single vs multiple), number of Win States (single win state vs multiple win states), Type of Opponent featured by the game (active opponent vs passive threshold), the presence of Time Pressure (present vs absent), and the Primary Interaction method of the game (combat vs noncombat) were chosen, ideally those choice can be tied to cognitive processes that are assumed to be affected by the engagement in playing those games. Finally, I wondered whether movement style and perspective code different factors. Thus, did the authors correlate their game factors or run a cluster/factor analysis to uncover shared and unique variance contributed by those factors.*
   a. Justification for why each gameplay factor was chosen has been added to the methods section (line 148-164), and a specific call to refine the game factors approach with respect to specific cognitive demands has been added to the discussion (lines 614-617). We agree with reviewer one that establishing how specific gameplay factors influence specific cognitive demands would be a major boon to the cognitive science of video games and VGT, though establishing that experimentally would first require a standardized approach to categorizing gameplay which doesn’t exist within the field at the moment – and which this meta-analysis is intended to facilitate.
   b. Re. movement style and perspective: Perspective codes weather the player perspective is from within an actual or implied player avatar (1^st^-person) or if that avatar is visible to the player (3^rd^-person). These factors are correlated (see the additional correlation analyses added to the results section, lines 287-290 & Table 2) but are not redundant.
      i. For more concrete examples, the present analysis included 1^st^-person games with egocentric movement (i.e. *Call of Duty, Unreal Tournament, Portal*), 3^rd^-person games with egocentric movement (i.e. *Grand Theft Auto V, Mario Kart, World of Warcraft*) and 3^rd^-person games with allocentric movement (i.e. *Pac-man, StarCraft, The Sims*). It does not appear that 1^st^-person games with allocentric movement have yet been studied within the field, though such games do exist (see the examples given on lines 512-513, all of which feature first-person perspectives coupled with allocentric map-based navigation, i.e. the player can chose their first-person vantage point by selecting that point from a list or an allocentrically-presented map).
4. *the authors did not motivate their choice for the outcome measures either or do give any examples (what is an attention/perception outcome and why is it not a HC outcome).*
   a. Further justification for the construction of our outcome constructs have been added to lines 229-244

­­­­­___________________________________________________________________________________

**Reviewer 2 Comments:**

1. *Confounds. The first relates to the interpretation and presence of confounds. As is now standard in intervention studies, the use of a control group is essential to rule out potential confounds, such as placebo, test-retest or practice effects. This is also true in meta-analyses that aim to compare different groups or interventions.*
   a. The reviewer is correct that utilizing a control group is essential for controlling for many sources of noise in a study, including in meta-analyses. Ideally, we would have calculated a relative gain measure for all examined interventions versus an active control condition. However, considering that the following:
      i. A substantial portion of the examined publications utilized video games as an active control group (i.e., Belchior et al., 2013; Blacker et al., 2014; Clemenson & Stark, 2015; Cohen, Green, & Bavelier, 2008; De Lisi & Wolfard, 2002; Feng, Spence, & Pratt, 2007, etc.), or examined differences in transfer across multiple video games without specifying one specifically as a control condition (i.e., Adams, 2013; Bailey & West, 2013; Boot et al., 2008; Glass, Maddox, & Love; 2007; Gonzales, 2012; Huang, 2020; Oei & Patterson, 2013; 2014a; 2015; etc.)
      ii. Video games utilized as a control condition must have a different gameplay profile to serve as a suitable active control to a video game intervention of interest
      iii. Creating a differential effect score comparing relative gain on two different games with two different profiles of gameplay features *necessarily confounds* the distinct gameplay features of the two games compared
   b. Considering the above, it is not possible the primary experimental question of this review – that is examining the differential effects of gameplay factors on cognitive training – using differential measures which necessarily confound different gameplay features. This is a limitation, but a necessary one to address the issue at hand.
   c. The mixed meta-regression approach presented in this re-submitted manuscript (lines 253-260, Tables 3-7) does not account for common confounds with regards to our estimation of the overall effect of game training, but it does allow us to definitively compare the effectiveness of subcategories defined by the coded game factors (i.e. combat vs noncombat games, etc.) which partially addresses this concern.
2. *Between group comparisons. Second, the use of within-subject effects also makes between-group comparisons less reliable as the Q statistics controls for only one source of heterogeneity related to within-group variance and not for the variance of between-group differences in pre-test performance, post-test performance or change scores, that are essential for comparing subgroups.*
   a. The mixed meta-regression approach presented in this re-submitted manuscript (lines 249-252, Tables 3-7) allows for direct subgroup comparisons. The discussion has been re-written to reflect the findings of these direct comparisons.
3. *Pre-post correlation. A third problem arises from the correlation between pre-test and post-test which is almost never reported in primary studies and thus calls for adjustments of the effect sizes and their variance. While assuming a prepost correlation of r = 0.5 is a neutral (i.e., unbiased) choice, this choice will impact the estimates, their variance (hence the weights) and thereby the results. It would be reassuring to see sensitivity analyses testing the impact of different values of r and this would certainly strengthen the results and conclusions. This is particularly important given that the meta-analysis is based on within-subject change scores and the correlation between pretest and postest can thus be expected to be higher than 0.5. Yet, sensitivity analyses testing several values below and above 0.5 would seem advisable.*
   a. While a sensitivity analysis would allow for the assessment of how different estimates of *r* would affect the results, it wouldn’t allow for a more accurate estimation of the “correct” estimate of *r* for pre-post correlations in this sample. Yes, we could assume that pre-post scores are highly correlated due to the within-subjects nature of the comparison, but that assumption is impossible to confirm with the data available. We thought it prudent to select a single unbiased value for *r* (=.5, which the reviewer noted as well) and run the analysis only once, rather than select between *r* values based on patterns of results.
4. *Random effects model. The authors use random effects models, which assume dependent effect sizes. Yet, the shared excel files (which are hardly readable and would be much more useful as downloadable excel files) suggest that the authors extracted more than one effect size from each study and group of participants. Were effect sizes coming from the same study aggregated or randomly selected and how? If averaged, how was variance handled? If randomly selected, did the author assess sensitivity via bootstrapping? Because effect sizes from the same study may not be fully independent (even if coming from different groups), an alternative may be to use methods that can handle dependent effect sizes, such as multilevel methods or meta-analytic models using robust variance estimates which allow to specify a correlated or hierarchical structure of the weights.*
   a. The “unreadability” of the shared excel file possibly suggests that the reviewer was viewing a preview of the file, not the file itself. We recommend downloading the file (upper-left corner on most browsers) which will grant the reviewer access to the excel file in full.
      i. Difficulty accessing the file (i.e. if there is no “download button) may be the result of security measures put in place by our parent university. We will happily supply this file directly (via the Editor to maintain anonymity) if the issue persists, but we believe we have set access permissions sufficiently to avoid this problem.
   b. Multiple interventions/groups from the same study were treated as separate studies in the initial analysis and a separate effect size was calculated for each, as specified on lines 224-228 of the manuscript.
   c. In line with this reviewer’s suggestion, we have taken a mixed effect meta-regression approach in the resubmitted manuscript, with the effect of study of origin modeled as a random effect in all analyses. Modeling the random effect by study rather than by intervention/group ensures that the inherent inter-relation of investigations arising from the same study is accounted for in the revised modeling.
5. *Moderators were assessed across multiple subgroup analyses. Were all analyses pre-registered and if yes did the authors consider correcting for multiple testing? One drawback of this approach is that it assesses the impact of each moderator individually*, *without controlling for other moderators, some of which may covary (e.g., genre, format, features, participants age). Meta-regression models can assess the influence of several moderators simultaneously. Meta-regression was used to assess continuous moderators and a similar approach may be recommended at least for some analyses of categorical (or dummy coded) moderators. Finally, some subgroup analyses may be underpowered as they rely on relatively small numbers of studies or effects.*
   a. As stated above, we have we have taken a mixed effect meta-regression approach in the resubmitted manuscript, in line with this reviewer’s suggestion. As suggested by this reviewer, these mixed meta-regression models include both the study and participant quality moderator as well as categorical moderators, allowing for the effects of each to be controlled for the others, and reducing the total number of comparisons substantially.
6. *Publication bias analyses are limited to trim and fill, which has been extensively criticized. There are a multitude of methods available now, each with its own pros and cons, that are generally recommended over the trim and fill approach. An increasingly common approach is to run publication bias sensitivity analyses using a variety of techniques (Carter et al., 2019; Mathur & VanderWeele, 2020). Moregenerall, I highly recommend these two recently published meta-analyses which provide state-of-the-art methods in terms of meta-analytic models for dependent effect sizes, moderator analysis and publication bias detection (Coles et al., 2019; Lehtonen et al., 2018).*
   a. We removed the trim-and-fill method as this reviewer suggested, and instead corrected for publication bias using a generalization of Egger’s regression test as explained on lines 307-310. This approach was chosen specifically because it could be integrated with the mixed effect meta-regression models used in the re-submitted manuscript.
   b. We agree that correcting for publication bias with regards to *p*-values rather than study variances, as suggested by Carte et al., 2019 and Mathur & VanderWeele 2020 is likely a more ecologically valid method of correcting for publication bias, and an approach that should be considered seriously by the field. However, as far as we are aware such methods have not been implemented in statistical packages in such a way that also allows for multi-level or mixed-effect meta-regression models (the approach taken in the re-submitted manuscript, also recommended by this reviewer in questions R2.4 and R2.5). Existing statistical packages (i.e. the “PublicationBias”, “puniform” packages for R) only allow for this method to be applied to simpler regression models. There is clearly a need for development of such a statistical package, but we unfortunately lack the coding expertise to develop such an approach in the timeframe of this review process.
7. *The introduction and discussion largely argue that the existing literature has emphasized an association between video game features and their cognitive effects and the present study attempts to address this hypothesis. Yet, I believe that the coding could be improved to better reflect the authors’ hypotheses as coding was sometimes inconsistent with the authors argument. Some games could be arguably coded in a different genre and some tasks in different constructs which might change the results and conclusions.*
   a. The coding of games into broad genres is problematic – even in the implementation we used in this meta-analysis – and this is a major impetus for conducting this meta-analysis, as discussed in the other responses to Reviewer 2.
   b. The justification for the coding of cognitive constructs is explained in our response to question R2.13
8. *Coding of game features. The part I liked most concerns the coding of game features which I found particularly interesting and could have been pushed even further. For example, a theory-grounded coding mapping specific game mechanics with particular cognitive demands (rather than mechanics associated with genres) would probably allow a different set of testable predictions.*
   a. We agree with reviewer 2 that the coding of game features can and should be further developed – one of the impetuses for this meta-analysis is to spur such future work. The authors assert that the field has yet to develop a thorough enough understanding of which gameplay features are associated with what cognitive capacities to allow for such a comprehensive categorization strategy, and that developing such a strategy as game cognition research continues to mature is of strong importance.
9. *Coding of game genre and format. What criteria were used to define genres and format? casual and serious games have been considered as video game genres in other studies, suggesting these 2 categories may overlap at least partially. Aren’t action, strategy, casual and serious meant to be mutually exclusive categories? How was the game Tetris (from Belchior et al) classified? It appears in both action and casual — which would seem wrong to me? How about Portal 2? Was it considered an action game and on which criteria? Were serious games only commercial games (how about cognitive training like cogmed, lumosity)? How about brain training games? Are these considered casual or serious? These notions should be clarified. Note that some codes (e.g. format, serious) were missing from the shared word doc and that the excel files were hardly readable or usable (no possibility to search). Also, the word “commercial games” is mentioned for the first time in the discussion line 453. Does that refer to off-the-shelf games? Was that an inclusion criterion? Were all serious games commercial games? There have been debates and inconsistencies in how genre was defined and handled in prior meta-analyses (either as selection/inclusion criteria or as moderator) and I am not clear if and how format differs from genre. This is a central point of the manuscripts that deserved some clarification including whether and why these codes are considered independent.*
   a. Our response to this question has been organized into multple parts to address the multiple issues that Reviewer 2 raised in this comment
      i. “*What criteria were used to define genres and format?”*
         1. The distinction between “Genre” (action|strategy) and “Format” (“serious|casual”) was based largely on work by Banequed and colleagues (Banequed et al., 2013; 2014; Smith et al., 2020), which demonstrates that “casual” video games fall into the same genre distinctions (i.e. “action, strategy”) as their non-casual counterparts. We therefore categorized both serious and casual games as either action or strategy. This is specified on lines 130-132 of the Methods section.
      ii. *“casual and serious games have been considered as video game genres in other studies, suggesting these 2 categories may overlap at least partially. Aren’t action, strategy, casual and serious meant to be mutually exclusive categories?”*
          1. *“*Action” and “Strategy” are mutually exclusive in our coding strategy, as are “Serious” and “Casual”. However, all games are categorized on both of these variables (so a game may be “Action” and “Serious”, “Action” and “Casual”, and so on).
          2. “Serious” in the context of this meta-analysis simply refers to a game that is not a “casual” game (as defined on line 131 of the methods section) and therefore these are mutually exclusive designations
      iii. *“How was the game Tetris (from Belchior et al) classified? It appears in both action and casual — which would seem wrong to me?”*
           1. Both *Tetris* and *Super Tetris* were categorized as a “Casual" and “Strategy” games by our raters. According to supplementary table 1
           2. As discussed above, all games were categorized as either “Action” or “Strategy” and either “Casual” or “Serious” - this dual categorization is not an error.
      iv. *“How about Portal 2? Was it considered an action game and on which criteria?”*
          1. Both *Portal* and *Portal 2* were categorized as “Serious” and “Strategy” by our raters. Again, this dual categorization is intended
      v. *“Note that some codes (e.g. format, serious) were missing from the shared word doc and that the excel files were hardly readable or usable (no possibility to search).”*
         1. The “Serious” vs “Casual” distinction (I.e. *Format)* was original summarized by the “Casual Game” column of Supplementary Table 1. This has been re-labeled as the “Format” column, with each game listed as “C” (Casual) or “S” (Serious) to address this confusion
         2. The inability to zoom or search on the shared excel file was an unintentional effect of the security protocols used in UTD’s file sharing service. We have taken steps to remedy this. If difficulty accessing this file continues, we would happily provide a copy directly (via the Editor, to preserve anonymity)
      vi. *“Also, the word “commercial games” is mentioned for the first time in the discussion line 453. Does that refer to off-the-shelf games? Was that an inclusion criterion? Were all serious games commercial games?”*
          1. The “commercial games” wording was an artifact of a previous draft of the discussion section which was erroneously retained in a submitted draft. Third has been corrected
      vii. *“Were serious games only commercial games (how about cognitive training like cogmed, lumosity)? How about brain training games? Are these considered casual or serious? These notions should be clarified.”*
           1. We did not examine cognitive training or brain training games in this meta-analysis, as we feel that cognitive impact of those games is qualitatively different than games meant as entertainment and therefore not directly comparable. This has been specified on lines 117-120.
      viii. *“There have been debates and inconsistencies in how genre was defined and handled in prior meta-analyses (either as selection/inclusion criteria or as moderator) and I am not clear if and how format differs from genre. This is a central point of the manuscripts that deserved some clarification including whether and why these codes are considered independent.”*
            1. The authors strongly agree with Reviewer 2’s statement here, and hope that the responses included in this document and changes to the manuscript have clarified this issue.
10. *Also, the genres Action and Strategy may be (arguably) too restrictive to capture all games. Forcing all games to fit into these 2 genres results in categories that are too heterogeneous to be meaningful. For example, the Action genre may not be the best fit for games like Centipede, Donkey Kong, Pacman or Wii Fit… Similarly, games like Angry Birds, The Sims or Tetris do not seem to fit well into the Strategy genre. Others have considered categories like simulation, exergame or platform games or even brain training. The literature search mentions brain training games but I didn’t see any. Were brain training games excluded and why? And if not how were these games coded?*
    a. Indeed, the Action/Strategy distinction is inadequate to categorize all games (the authors opinions on the topic broadly align with Dale & Green, 2017, as indicated in lines 48-53 of the introduction).
    b. The inclusion of the Action/Strategy coding scheme alongside the more detailed Game Factors coding approach was designed contrast (one of) the ways Genre has been classified by the field in the past with our newer approach. We do not endorse the Action/Strategy distinction, and as Reviewer 2 notes we argue against the utility of that classification strategy at several points. Thus, the authors observe no contradiction in its use in this study as a point of comparison to the more detailed classification method we propose here.
    c. The distinction between simulation, strategy, platforming, and action games is poorly defined throughout the literature, as discussed extensively in our introduction (lines 54-84). Our argument is that any such genre distinctions are by-and-large arbitrary and unhelpful – therefore the two-genre action/strategy categorization approach is just as valid (or invalid) as a 4-genre approach which also includes puzzle or platformer games.
    d. Related to the above, we instructed our reviewers to categorize games as “action” or “strategy” as they deemed fit – this was an enforced binary choice.
    e. Exergames and brain training games were excluded from the present analysis. This has been specified on lines 116-122
    f. Related to the specific example cited by Reviewer 2: according to supplementary table 2, *Angry Birds, Centepede, Donkey Kong, Pac Man,* and *Pac Man: Adventures in Time ,* were coded as “action” games by our raters, while *The Sims, The Sims 2, The Sims 3, Tetris,* and *Super Tetris* were all coded as “strategy” games by our raters. Wii Fit, being an exergame, was excluded from the present analysis in line with the above comment.
11. *More importantly, the categorization of games into genres seems inconsistent with the authors' argument that games that belong to a given genre share features that are responsible for certain cognitive effects.*
    a. See response b to comment R2.10
12. *Coding of experimental and control games. Here too, the reader would benefit from a more detailed description of how this coding was performed as it wasn’t available in the supplementary excel file. Note that the coding of serious games is also missing… Again, these are important as the criteria for defining serious games or experimental vs control games has varied across studies.*
    a. Games were not universally coded as “experimental” vs “control” - this was a binary factor of the intervention examining a game, not of the game itself, as specified on lines 206-208 of the manuscript. If the condition from which an effect size was generated was an experimental condition in its study of origin, that case was labeled as an “experimental” study (see supplementary table 2). Similarly, if the condition from which an effect size was generated was a control condition in its study of origin, that case was labeled as an “experimental” study (again, see supplementary table 2)
13. *Coding of cognitive constructs. Relatedly, a similar reflection would also be relevant for defining cognitive constructs of interest. What criteria guided the choice of these 3 outcome categories: attention/perception, higher cognition and psychosocial? Previous meta-analyses of video games (and in other fields too) have categorized cognitive measures into separable constructs may provide some guidance or inspiration (e.g. Bediou et al., 2018; Powers et al., 2013; Powers & Brooks, 2014; Sala et al., 2018; Wang et al., 2017). What is the rationale for including some memory (working memory) tasks in higher cognition, whereas others are coded as memory? Psychosocial also includes depression and anxiety scales, affect or emotion (PANAS), risk taking… which may not really correspond to psychosocial skills. Again, the categories appear too heterogeneous to be meaningful. For example, memory may include tasks that include manipulation of verbal or visuo spatial material which may be differentially impacted by distinct games. Studies of memory may also include older participants and although the effect is NS, the trend is negative suggesting smaller effects in older participants (consistent with previous meta-analyses that treated age as categorical that report smaller effects in older adults).*
    a. Further justification for the construction of our outcome constructs have been added to lines 229-244. Working memory was specifically coded as Higher Cognition and not memory because of its known inter-relation with reasoning and executive function (see Miyake et al., 2020)
14. *The authors conclude that game features predict differential effects on cognition. Yet, their effect sizes do not differentiate between skills as the confidence intervals overlap and the use of within-subject effects does not allow proper between-group comparisons. Therefore, some conclusions may need to be toned down or rephrased and these limitations should be better acknowledged.*
    a. As stated above, our revisited statistical approach does allow for direct subgroup comparisons. The discussion section has been re-written with respect to only those differences found to be significant with the revised approach to analysis.
15. *Mapping between features and cognitive constructs. The game features coded do not clearly map onto specific cognitive demands, which appears to contradict what the authors discuss about the choice of experimental and control groups in primary studies. Lines 465-477, “Importantly, “control” condition games failed to produce any significant transfer to any cognitive construct, despite a diverse range of games being presented as active control conditions (see Supplementary Table 1). This is evidence that, in general, VG-based interventions have tailored their cognitive transfer measures specifically to be sensitive to the cognitive demands of the intervention, and/or have intentionally selected control games not expected to produce transfer to the constructs examined. While this is an understandable design strategy, this practice may lead authors to incorrectly conclude that the control games used, or aspects thereof, do not produce cognitive improvements. As a concrete example of why this is problematic, consider the games Tetris and The Sims. Both games have been frequently used as active control conditions in studies of “action” video games, and producing negligible transfer as expected [12, 13, 37]. However, both of those games have demonstrated significant transfer, in studies in which they were an intervention of interest.”*
    a. This statement makes no claims as to the link between cognitive constructs and specific game features – the lack of transfer to *ANY* cognitive outcomes from Control games (which, as established by this meta-analysis, have a multitude of gameplay profiles spanning the coded gameplay features) preclude gameplay features as an explanatory factor of this lack of transfer. We suggest that a form of selection bias is present, in which experimenters are selecting control games which they believe a-priory will not cause transfer to their outcome measures.
    b. This statement has been re-worded in light of this comment and the findings from our adjusted statistical approach (see lines 473-488)
16. *The cases of Tetris and The Sims are particularly illustrative and interesting as the choice of using these as experimental or control games may depend on which aspect of cognition was measured (and considered as demonstrating transfer). This seems to contradict another statement that appears a few lines later. Lines 500-510: “the above definition of “action” games would translate to games that are combat-focused with time pressure, and have an active opponent. However, the current meta-analysis found consistent transfer to measures of attention and perception was facilitated by games that featured a first-person perspective, a single controllable object, a single win state, lacked an active opponent, and were primarily non-combat games. Not only was transfer to the attention and perception construct found to be agnostic to the presence of time pressure, but both active opponents and combat gameplay explicitly failed to produce consistent transfer. This finding provides evidence that the post-hoc justifications given in the past explaining the link between “action” video game play and enhanced attention and perception may be inaccurate.”*
    a. The referenced statement has been substantially altered in light of the findings from our adjusted statistical approach as well as this comment (see lines 519-534)
17. *These are strong statements and conclusions which depend largely on the appropriate coding of game features, video game genres and cognitive construct, as well as their appropriate analysis allowing more direct comparisons which were not possible here.*
    a. As stated above, our statistical approach has been substantially altered and now does allow for direct subgroup comparison – the resubmitted discussion is written with regards to the findings of this updated statistical approach
18. *Finally, the sentence line 566-567: “The gameplay factors analysis conducted in this meta-analysis rebuffs the “action” vs “strategy” distinction as useful.” appears too strong and should be downplayed given the results and the rest of the discussion arguing that this distinction has some value. This study sheds some light on how some of the game features that are present in action and/or strategy games and may be associated with their (differential?) cognitive effects. However, I don’t think it rebuffs the distinction between action and strategy games… As the authors rightfully discuss on line 573, the “genre distinction does have merit” (as well as some “historical” value) as action and strategy games were initially quite distinct in terms of mechanics, features and cognitive demands, and thus represented meaningful categories less than 10 years ago. However, as the video game ecosystem grows and new genres, hybrid genres and mixed genres arise, the features that were once specific to action or strategy games have become more and more common across genres and thus more difficult to isolate (see (Dale et al., 2020; Dale & Green, 2017). Toning down the conclusion won’t minimize the potential impact of this study which will make an important contribution to the field and should stimulate more work looking at how game characteristics map onto cognitive demands and whether they predict cognitive effects.*
    a. The conclusion re the utility of genre distinction has been re-worded in light of this comment to be less definitive and more accurately reflect the limitations of this work (see lines 580-585). We should note that the evidence supporting the partial merit of genre distinction was in fact weakened by our adjusted statistical approach, which did impact our interpretation of these results.
19. *The authors argue that the lack of effect in the control condition “is evidence that, in general, VG-based interventions have tailored their cognitive transfer measures specifically to be sensitive to the cognitive demands of the intervention.” (line 468). This conclusion would require an analysis based on the cognitive demands rather than the game characteristics –which were not coded here– unless there is a perfect match between game features (e.g. pace or time pressure) and cognitive demands (attentional control)?*
    a. Our argument re the possible influence of selection bias on the relative efficacy of experimental vs control condition games has been re-worked, see lines 470-484
20. *Study quality / risk of bias. Details about the PEDro scale would be appreciated, especially because it is less commonly used than other tools such as the Cochrane risk of bias scale. (https://guides.himmelfarb.gwu.edu/systematic_review/reporting-quality-risk-of-bias).*
    a. More information regarding the PEDro scale as well as justification for its use have been added in lines 134-138
21. *Participant and study characteristics. Meta-regressions show non-significant modulation by either participant or study characteristics. Were these moderating influences also considered in subgroup analyses? Some factors tend to covary (e.g., sample size, age, training duration) and may thus be difficult to disentangle.*
    a. The participant and study characteristics were not considered in the subgroup analyses presented in the original submission, but are included in the mixed effect meta-regressions in the revised manuscript.
22. *Participants characteristics: Are groups matched in gender? Proportion (%) females overall can be misleading if the groups are not balanced. Why was average age treated as continuous… previous work has mostly used age-group categories (e.g., children, young adults, old adults).*
    a. The majority of studies examined either reported no difference in gender between experimental groups or explicitly gender matched the groups (see table 2). Some had disproportionately more males across all groups (i.e. Adams 2013, Di Lisi & Cammarano 1996), and some explicitly compared the effects of training between genders (i.e. Feng, Spence & Pratt, 2007; Subrahmanyam & Greenfield, 1994). This disparity between studies, and in some cases between groups in the same study, is why we included % female as a moderator of interest.
    b. Average age was treated as continuous for several reasons. Firstly, age bin cut points are not consistent across the field (i.e. some studies may categorize participants 60 and older as “older adults”, some 65 and older, etc.). Second, even if age bit cut points are consistent, there are considerable differences in cognitive profile and social cohort between individuals in the same age bin – i.e. an 80-year-old “older adult” would be expected to have a substantially different cognitive profile than a 60-year-old “older adult”, while a 20-year-old “younger adult” would be of a substantially different social cohort compared to a 40-year-old “older adult” especially with regard to video games and technology. Using average age as a continuous variable allows us to preserve variance that may correspond to these differences.
23. *Study characteristics: Number of participants is already included in the weights (inverse variance) and therefore I don’t think it needs to be added to the models. What was the rationale for coding the number of cognitive outcomes? What result was expected? It is surprising that total hours had no effect but again there may be complex interactions with participant characteristics, cognitive construct, game features or genres.*
    a. Re number of participants: this point is well taken - *n* was not included in the meta-regression models due to the noted redundancy with the weights used.
    b. Re number of cognitive outcomes: Since a single effect size of each construct was calculated from each study regardless of the number of outcome measures, controlling for that number accounts for systematic differences in effect size that this variance of measures may induce, such as relative susceptibility of each effect size to the task impurity problem (see Miyake et al., 2000). Previous work from our group has found such systematic differences (Basak, Qin & O’Connel, 2020).
    c. Re total hours of training: The submitted manuscript first meta-analysis to show negligible gains from longer training durations – see Basak, Qin, & O’Connel (2020), Toril, Reales, & Ballesteros (2014), Li et al., (2011) for other examples. Aggregate evidence seems to point towards diminished returns after about five hours of training.
