# Supplementary material for: A game-factors approach to cognitive benefits from video-game training: A meta-analysis
Source: PLoS One. 2023 Aug 2;18(8):e0285925. doi: 10.1371/journal.pone.0285925 (PMC10395941; doi:10.1371/journal.pone.0285925)
Supplement: Attachment — Submitted filename: Smith & Basak 2022 Response to Reviewers 03.29.2023.docx [file pone.0285925.s009.docx]

**The reviewers’ comments are in italics. The response are below the comments.**

**Reviewer #1’s Comment*:*** *First I would like to thank the authors for addressing my former remarks. I found the manuscript much improved and could find any information I looked for. However, I am still a bit confused regarding the Overall Cognition Factor, what does this mean and does the fact that also all other outcomes are then analysed. In my view the Overall Cognition effect sizes needs better motivation or should be left out.*

a. The authors have revised the manuscript w.r.t. to overall cognition. We believe that in line with the pre-registration, standard reporting of cognitive training on cognition, and past mixed results on videogame interventions effects on cognition, the reporting of overall cognition is important and has a strong value, esp. where the moderator effects are observed.

**Reviewer #2 Comments**: *I would like to reiterate my strong interest in the approach and results. I sincerely believe the analysis of video game features is a great and promising improvement over the game genres approach, and a timely one as the early conceptualization of video game genres needs to be reconsidered to reflect the diversification of video game genres (and of the whole video game ecosystem). Concerning the revised manuscript, the authors have properly addressed most of my concerns, especially regarding the meta-regression analysis. However, there are a few conceptual (theoretical) as well as methodological issues that remain unresolved and that, once clarified, could improve the paper’s impact.*

**Main Comments:**

1. *First and foremost, I think the coding of game genres and format could be improved. The dichotomic conceptualisation of genres and formats is not only inconsistent with a growing literature on the evolution of video games reviewed in the introduction (lines 47-48): “a coarse distinction between gaming genres is insufficient to describe the profile of cognitive demands of a given game, as modern video games increasingly include features of multiple genres [7].” , but also, and more problematically, the chosen categories for genres (Action vs Strategy) and formats (Casual vs Serious) do not align with the existing literature and with the evolution of the field over the past decades (e.g., Dale et al., 2020; Dale & Green, 2017). Forcing all games to fit into these 2x2 categories is likely to result in blurred and overlapping categories with high heterogeneity, which could account for the lack of significant effects of these 2 factors. To illustrate my point, I was surprised to see games like “Tetris” or “Wii Fit Segway Circuit” be classified as Action-Casual. In contrast, games most commonly associated with the action genre, which are mostly first person shooter games, were found in the Action-Serious category which also included games like Fifa or super mario or pac-man. Such coding is only likely to add to the confusion the authors initially denounce in their introduction (e.g., note here that “Tetris” also appears in the strategy-casual category).*
   a. The authors entirely agree with reviewer 2 that these binarized genre classifications are defined on inherently indistinct categories, and have likely resulted in very heterogeneous games being classified within a given category. We disagree however that these distinctions “*do not align with the existing literature”*. Our selection of “action” vs “strategy” and “casual” vs “serious/non-casual” was primarily based on Simons et al., 2016’s review of video-game training literature, which as reviewer 2 alluded to identified “Action”, “Strategy”, and “Casual” as the most common labels applied in the literature at the time. While the cited papers by Dale and colleagues rightly assert the limited utility of genre classification, we assert that the vast majority of the literature still utilizes the genre classifications that we have binaries and encoded in this meta-analysis, particularly as the majority of the studies we cite were published before the Dale articles. In short, we agree that these categories do not properly reflect meaningful differences in gameplay profiles, but *do accurately reflect how these terms are (mis)applied in the bulk of the literature we are reviewing*, and hence serve as a meaningful “control” to the game factors approach we recommend.
   b. Re the examples of Tetris and Segway Circuit
      i. “Tetris” and “Super Tetris” were both categorized as strategy-casual by our raters (see supplementary table 1 and our shared dataset). The only reference to “Tetris” in relation to “action” games as far as the authors are aware is the mention that “Tetris” is often used as an active control game in action game interventions (alongside “The Sims”).
      ii. In terms of gameplay, Segway Circuit is a time trial racing game, strongly resembling other racing games which have been lumped under the “action” game umbrella such as “Need for Speed”, “Gran Turismo”, and “Mario Kart”, with the major difference that those games may (but don’t necessarily, based on the game mode selected) feature active opponents. In our opinion, it is not surprising that the majority of our raters coded “Segway Circuit” as an “Action” game based on these characteristics.
2. *Second, the discussion relies heavily on non-significant effects, which should not be interpreted as evidence for the absence of a difference. For example, the non-significant effect of game genre (and format) may not be surprising considering how games genres and formats were coded in this meta-analysis – which deviates from the definitions of genre and format used in primary studies and in other meta-analyses too. More importantly, the authors rely on the lack of significant effects to criticize and thus diminish the importance of earlier work conducted more than 1 or 2 decades ago, when only a small number of genres were sufficient to appropriately capture meaningful differences between video games in terms of mechanics or gameplay features (and their associated cognitive demands).*
   a. This is a fair criticism, and our discussion has been revised to more soberly interpret these non-significant findings (as further specified in comments below).
   b. As an aside, we disagree with the assertion that “*only a small number of genres were sufficient to appropriately capture meaningful differences between video games... one or two decades ago”*. While we go into some depth re. this opinion in other specific responses (#13, among others), fully addressing this is outside the scope of these responses. The primary author (Evan T. Smith) would happily continue this discussion with reviewer 2 after the review process is complete, should they wish to do so.
3. *In sum, the analysis of game genre and format needs to be improved. At the very minimum, the authors need to fully address the limitations of their choice of grouping and categories and how it is inconsistent with previous work. Alternatively, given the confusion it is likely to add, this analysis could be dropped., In all cases, the discussion should be significantly altered to (i) not over-interpret non-significant effects and (ii) acknowledge that the 2x2 coding approach taken in this work does not reflect the complexity of the video game ecosystem to date and, for the most part, does not align with the categorization of games used in primary studies.*
   a. The limitations of our approach have been more strongly emphasized in our discussion, as detailed in later comments. In regards to dropping this analysis, we feel, as elaborated in our response to comment #1, that our coding of genre does reflect how genre has been (mis)applied in the bulk of past literature, and feel it is therefore a valuable inclusion in this manuscript
4. *I particularly liked the idea of analyzing video game features, which is the main strength of this study, although the pattern of inter-correlations calls for a cautious interpretation of the results. This actually raised the question of how were these correlations computed since all variables are coded as binary (0 or 1)? What tests were conducted and what type of correlation coefficients are reported in Table 2?*
   a. Spearman’s correlation coefficient is reported on Table 2 (though in the case of binarized data that is equivalent to Pearson’s or Kendal’s tests). This is admittedly not the most rigorous way of assessing patterns of inter-correlations, but it does convey the fact that these factors are inter-related in a complex way, which as reviewer 2 mentions is important in interpreting our results.
   b. As with the coded game factors themselves, a more rigorous mapping of how game factors relate to one another is a next logical step in this line of research, which the authors are keen to pursue in future manuscripts.
5. *While these results are an important step forward, they do not necessarily invalidate past research, unlike what the authors suggest. Indeed, many of that past research was conducted at a time when games could still be unanimously classified into a relatively small number of distinct and homogeneous genres.*
   a. See response to comments 2 and 11.
6. *In this respect, including the variable “study group” in the analysis of game features, as well as in the analysis of genre and format seems an unmotivated choice. More generally, it is unclear how genre, format and group are related to game features. Alos, what was the rationale for including study group only and not genre and format in the analysis of game features? Are these three factors correlated and how do they correlate with game features? Adding all 3 variables to Table 2 and showing how strongly video game genre, video game format and study group correlate with game features would be essential to better understand and interpret the pattern of effects (see point 3).*
   a. Regarding the relationship between genre, format, and game features – indeed we agree that the relationship of specific game features to these commonly-used genre distinctions are indistinct and by-and-large arbitrary, which is why we propose the game factors approach as a more viable alternative.
   b. Regarding the logic of excluding genre and formant form the game factors model – we conceptualize the game factors approach and the genre approach as two different ways of categorizing the same complex phenomenon – that is the wide range of possible gameplay styles present in commercial video games. We’re not interested in seeing how gameplay factors relate to cognition above-and-beyond genre – we’re interested in which of these methods more accurately relate to cognitive outcomes of training. Hence, we ran models featuring each approach separately, and then compared model validity between them (via AIC in this case).
   c. Genre/Format have been added to Table 2.
7. *The discussion of the associations between individual game features and cognitive outcomes suggests that some earlier theories may have been misinterpreted. On multiple occasions, the authors misreport the action games literature as predicting that specific features are responsible for their cognitive effects. My reading of this literature is that it has put the emphasis on identifying the combination of features that distinguishes action games from other games that do not produce similar cognitive benefits. This question could be addressed by computing the similarity between games across a number of critical features such as those coded here. Recent theories actually propose that the effects of action games on cognition arise from the particular combination of features that characterize these games (including those coded here and many others such as the presence of variable rewards, the scaffolding of difficulty and challenge to keep players in their zone of proximal development, etc.). Importantly, the action video game literature does not predict that each and every feature in isolation has an effect, quite on the contrary (Bavelier & Green, 2019; Cardoso-Leite, P. et al., 2020). “Crucially, it has been our experience that each of these three characteristics on its own does not guarantee cognitive impact, at least when it comes to attentional control enhancements. Rather, action video games are unique in that they naturally layer these three game characteristics within the same overarching game play. For example, games that put a premium on just one characteristic such as pacing do not seem to similarly enhance attentional control and other aspects of cognition (e.g., Tetris).” (Green & Bavelier 2019, page 156).*
   a. We thank reviewer 2 for this valuable input, and have adjusted our discussion accordingly (see comments below), and specifically invoke the idea that our analysis of specific game features does not account for their conjunctive effects (i.e. lines 509-516, 545, 620-621)
8. *This misreading of the literature is only briefly mentioned in the discussion, lines 581-584 (typo included): “Importantly, of the central predictions of the originating from the action game training literature – that combat-focused games facilitate cognitive transfer to both attention/perception and higher-order cognitive functions [1, 25] – is in agreement with the findings of the current meta-analysis.” This is not only a misinterpretation of the action game training literature that has not claimed such type of feature-specific effect on cognition, but also a misleading terminology to reduce action games to combat-games – e.g., fighting games are not expected to have the same impact as action-shooter games. Moreover, the next sentence further highlights inconsistencies between the interpretation of game features and the fact that the adopted coding of game genres is highly uncommon (see also point 1): Lines 484-588: “However, 50% of the “action” games sampled in this study did not feature combat as a primary gameplay mechanism, whereas 40% of the examined “strategy” games did feature combat as a primary interaction, meaning that the finding that combat games facilitate transfer to both of these cognitive outcomes is not a genre-dependent one.” Taken together, these two statements illustrate a main problem with this paper which needs to be addressed: that the coding of game genres applied in this meta-analysis does not align with that used so far in the field.*
   a. As stated in comment 1 and elsewhere in these responses, we disagree that the coding of game genres differs from what is used I the field, though certainly not all publications conform to this action/strategy or action/nonaction dichotomy. That being said, the point re conflating action games with combat is well-taken. We have modified several sections throughout the manuscript to correct this (i.e. lines 600-604 & those sections mentioned in reviewer comment #18)
9. *The effect of “study group” is interesting, although not unexpected and consistent with prior meta-analyses focusing on video game interventions with active control groups. However, it remains unclear why this effect is indicative of the presence of a selection bias in past research and may thus contribute to publication bias (lines 466-484). The finding of stronger effect sizes in the experimental group is expected and doesn’t mean that the choice of control groups was intentionally biased (which the authors seem to interpret as a form of questionable research practice contributing to publication bias). There are two main problems with this claim. First, the present meta-analysis focuses on within-subject pre-post intervention effects, which are subject to important confounds (e.g., expectations, test-retest, practice or placebo effects), and are known to cause biases in meta-analytic investigations (e.g., see Cuijpers et al., 2017 for a detailed discussion of the limitations of this approach). I understand that this was necessary as the coding of game features required computing separate effect sizes for the experimental and control games. However, the limitations of this approach should be better acknowledged, especially given the strong interpretation the authors draw from the result. Second, the use of an active control group is critical for establishing causality and also necessary to rule-out non-specific effects and confounds. Importantly, the choice of control group is guided by the specific research question(s) addressed, which have evolved as the field has matured. To date, most active-control groups involve playing a commercially available video game in order to control for unspecific effects that may be induced by video game play per se (e.g., changes in affect or mood due to playing a video game for example). The choice of control games is thus driven to maximize across the dimensions or features (e.g., combinations of gameplay and mechanics) that are hypothesized to play a key role in driving the cognitive effect of the experimental game, while keeping equated important non-specific features (e.g., the very act of playing, positive feedback within the game, engagement with the training, social stimulation, etc). Despite a few exceptions in which the authors explicitly attempted to test whether the presence or absence of specific game features were critical (e.g., Oei & Patterson, 2015), the studies included in this meta-analysis did not seek to isolate a single video game feature, nor did they argue that a specific feature alone was causally responsible for the particular cognitive effects observed (see also Ben-Sadoun & Alvarez, 2022; Choi et al., 2020 for recent developments in this direction). In all, the discussion about selection bias reads as if the authors were assigning the wrong intention to the primary studies and thus reflects a misunderstanding of past research. To my knowledge, most studies included here sought to either replicate or clarify the extent of effects by delineating the particular types of games and cognitive domains impacted. To demonstrate the effectiveness of an intervention, primary studies assess whether greater benefits in the experimental group are found compared to the control group (the choice of control group is important to test hypotheses about the mechanisms of cognitive improvement). Tellingly, finding a larger effect in the experimental as compared to the control group does not mean that the control game did not produce any effect (as written line 479-480, but contradicted line 633-635) but instead that the effect of the experimental game was stronger than that of the control game. This whole section needs to be clarified to avoid misleading conclusions.*
   a. We thank reviewer 2 for this thorough and fair critique of our conclusions re. the experimental/control group findings. As with most of our discussion, we have reworked the sections in question to more accurately reflect the ambiguity in these findings that Reviewer 2 has highlighted here (see lines 482-495)
10. *The preregistration is lacking critical information about the analysis plan (meta-analytic models), hypotheses tested and expected results. This makes the preregistration less useful as it is not clear what represents a deviation from the planned protocol and still leaves researchers enough degrees of freedom and flexibility in the analysis and reporting of their results.*
    a. We thank the reviewer about this insight and opinion. At the time of pre-registration, the focus was mainly on the main aims regarding game factors meta and the inclusion/exclusion criterion. We therefore believe that the pre-registration is still important than non-registered meta-analysis in terms of the main goals of the project and the specifics of I/E criteria in the broad field of videogame.
11. *Genre (action vs strategy) and Format (serious vs casual) are first introduced as 4 types or categories of video games, and then analyzed as two separate and independent (orthogonal) factors. While I understand the rationale of casual games spanning various genres, I don’t understand the dichotomy between casual and serious and what was predicted. Here, only the serious-FPS category seems to correspond to what has been called action video games in past research and contains mostly first and third person shooters as typically used in action games interventions (which would show here as an interaction effect). A consequence of this dichotomous view of game genres is that some games do not fit in their assigned category. For example, the games Tetris, Wii Fit Segway Circuit, Angry Birds, Balance, Centipede, Pacman, marble madness, FIFA 2010, Pinball Hall of Fame, MultiTask, or New Super Mario Bros are labeled as action games despite being reported as non-action (mostly control) games in the primary studies. This is at best confusing and at worse likely to set the field on the wrong path.*
    a. The *format* variable is fairly rigidly defined via the definition of “casual” games commonly used in the literature, that being games designed to be played for 30 minutes or less in a single session (and “serous”/”long-form” therefore being designed to be played for longer than 30 minutes in a session). We agree, as mentioned in previous comments, that binarizing the action/strategy distinction does not adequately categorize all games, but as discussed in our response to comment #1 we feel this forces categorization accurately reflects the common misapplication of genre terms in the literature. Accurately categorizing the genre of a given game would require a much more granular approach than has been applied in the field to date, and in the end many of those distinctions would be arbitrary (do first-person shooter game and a first-person action game that does not involve projectile combat meaningfully differ in terms of cognitive demands evoked?). The authors assert that a more granular categorization of genre (i.e. “action/strategy/puzzle”) does not address the issues that reviewer 2 raises, whereas a game factors approach does – indeed, that is the thesis of this paper.
12. *I also wonder whether genre and format are meant to be orthogonal dimensions or simply different attributes. The classification proposed by Simons only considers 3 categories: action, strategy and casual. While I understand the authors’ argument for casual games that span distinct genres, I don’t think this applies to serious games --a term that has been more commonly used to refer to educational or therapeutic games, in contrast to entertainment video games. And indeed calling commercially available FPS or TPS, serious games is likely to be highly confusing to all readers.*
    a. We had intended the term “Serious” to refer to the inverse of “Casual”, i.e. games designed to be played for sessions longer than 30 minutes. We have adjusted this term to “long-form” throughout the manuscript, to avoid confusion with the term “serious” game as it has been used to refer to educational/therapeutic games.
13. *When the authors state that the genres have become more “blurry” (line 598), it would be helpful to clarify that this is due to both an increase in the number of genres and subgenres with the classification becoming more granular with more genres (rather than less) and narrower subgenres (e.g. FPS is a subgenre of action), together with greater overlap between genres and subgenres. This state of affairs has led to the emergence of hybrid-genres such as action-role-playing games that mix action mechanics* with *role playing features.*
    a. We don’t entirely agree that the in lack of distinction of game genres is resultant of *“both an increase in the number of genres … and narrower subgenres (e.g. FPS is a subgenre of action), together with greater overlap between genres and subgenres.”* Genres have always been a post-hoc categorization of a rich and complex gameplay space, from the earliest days of commercial gaming as a hobby. Stating that “genres are becoming more indistinct” miscategorized the issues – gaming enthusiast, marketers, etc. are continuously developing increasingly specific genre terminology to describe specific points in “gameplay space”, and admittedly this is driven by continual permutation of existing and development of new gameplay tropes in the developer space, but genres have *never* been accurate summaries of gameplay, (in this author’s opinion)
    b. A full exploration of this topic is, as mentioned, outside the scope of this paper (and indeed, the field of cognitive science) – considering this, we have elected to keep our summary of this issue brief, but have adjusted the wording to better convey the nuances of our argument beyond “blurry” (see lines 613-616).
14. *The analysis separating by outcome is interesting but may be underpowered and should thus be reported as exploratory or at least interpreted cautiously.*
    a. We acknowledge that our analysis of separate outcomes is lacking in power particularly with regards to memory and psychosocial outcomes on lines 570-580. We contend that the sub-analyses pertaining to Attention/Perception and Higher-order Cognition, with *k*s of 90 and 54 respectively, are sufficiently powered, particularly with respect to other meta-analyses.
15. *I found surprising that Movement and Perspective produce opposite effects in Table 3, given that they are strongly and positively correlated in Table 2. Could the author comment?*
    a. Correlation between the two predictor variables does not mean that they need to have the same main effect on the dependent variable, especially after properly controlling for other influences. As each fixed effect in the LME approach is corrected for all other fixed effects, we can state that, for example, the impact of *perspective* on cognitive transfer is negative, (i.e. favoring 1^st^-person perspective) *all other things being equal*, *including movement*. We would expect, based on these results, that both egocentric games in a 1^st^-person perspective would produce greater cognitive transfer than egocentric games in a 3^rd^-person perspective. The correlation simply states that, in addition to this, egocentric games are more likely to be 1^st^-person whereas allocentric games are more likely to be 3^rd^-person.
16. *I could not find the reference to Valdez 2011. Was this study only a 15 minutes intervention as suggested by the study label in the excel file (Experimental Study, 15 minute RDR-NV group)?*
    a. The full citations for studies included in the meta-analysis but not directly referenced in the body of the main manuscript can be found in our supplementary references, which for the Valdez study in question is “Valadez, J. J., & Ferguson, C. J. (2012). Just a game after all: Violent video game exposure and time spent playing effects on hostile feelings, depression, and visuospatial cognition. Computers in Human Behavior, 28(2), 608–616. https://doi.org/10.1016/j.chb.2011.11.006”. We note that the year of this study was incorrectly listed as “2011” in our public access data (this has been corrected), but is correctly cited to the year 2012 in the supplementary materials.
    b. To answer reviewer 2’s initial question, Valdez included both a 15-minute and a 45-minute intervention group. Indeed this is the shortest intervention included in this meta-analysis.
17. *Line 590: Did you mean coarse instead of course?*
    a. Indeed we did – we thank reviewer 2 for the correction.
18. *In conclusion, the present results do not support the following conclusions that(i) game genre is a useless construct; it has at least been valid and instrumental in guiding hypothesis generation and testing, (ii) the analysis of games features invalidates the theories about the action video game effects, (iii) the choice of control games reflects an experimenter bias (implying a form of questionable research practice). As a consequence, I would ask to rephrase and downplay several strong judgmental statements in the discussion:*
    i. We thank reviewer 2 for their thorough critique of our discussion, and recommendations for improvement. All of the sections listed below have been adjusted in line with reviewer twos feedback – line numbers after revision (as well as specific comments as necessary) are included below.
    b. *Lines 524-530: “However, games with passive thresholds or objectives better facilitate transfer to AP outcomes than did games with active opponents, and the AP construct was insensitive to the presence or absence of time pressure. This finding provides evidence that some of the post-hoc justifications given in the past explaining the link between “action” video game play and enhanced attention and perception may be inaccurate – specifically, it does not seem that active opponents or time pressure are necessary elements of gameplay to drive attention & perception outcomes.”*
       i. *This section has been reworked in line with reviewer 2’s suggestions (lines 538-545)*
    c. *Lines 630-640: “This analysis, in line with previous reviews [2] confirmed the presence of publication bias within the video game training field. Our finding with regards to transfer from experimental groups vs. active control groups using VG interventions may shed light on this problem. Based on our findings that videogames utilized in active control group failed to produce cognitive transfer regardless of their gameplay properties, we can reasonably conclude that some form of bias is suppressing significant transfer in those groups. Addressing this bias is imperative in its own right, and may contribute to the reduction of publication bias in future game training interventions. The authors suggest that future studies examine multiple games of various cognitive profiles with the assumption that differential transfer will be observed between groups (i.e. to differing measures of transfer), rather than the a-priori assumption that one condition will be less effective than another [15, 22, 23, 58].*
       i. *This paragraph has been removed. Discussion of selection bias is now relegated to lines 468-488 and presents, we believe, a more balanced perspective on the findings that intervention games consistently produced greater transfer than control games in this analysis.*
    d. *Lines 499-510: “While games utilizing these gameplay features are common in the field (“first-person-shooter” games utilize a first-person perspective, “real-time-strategy” and many “platformer” and “puzzle” games utilize allocentric movement), neither the first-person perspective nor allocentric movement style have been theorized to have a strong impact on cognitive transfer resulting from VGT interventions. Interestingly, no single game examined in the present meta- analysis featured both an allocentric movement style and a 1st-person perspective, though in theory these results suggest that a game featuring both would be a candidate for effective cognitive intervention.”*
       i. *This section has been reworked in accordance with reviewer 2’s comments (lines 505-524)*
    e. *Lines 549-552: “As was the case with transfer to the AP construct, transfer to the HC construct was insensitive to the presence or absence of time pressure, which has been invoked as a crucial factor driving cognitive transfer from both “Action” and “Strategy” game interventions [19, 25].”*
       i. *We contend that this statement re. the insensitivity of the HC construct to time pressure as well as the invocation of time pressure as a driver of cognitive change in past work is accurate. This has been reworded slightly so as to be less strongly assertive.*
    f. *Lines 576-581: “The findings of the present meta-analysis demonstrate the limited utility of broad genre classifications in understanding the effects of videogame training. Not only did the “action” vs “strategy” distinction prove ineffective in distinguishing cognitive outcomes of VGT-based cognitive interventions, the gameplay features where were found to differentially impact cognitive outcomes to general cognition, attention/perception, and higher-order cognition did not correspond to defining features of either genre.”*
       i. The “Relevance of Existing Genre Definitions…” (beginning on line 590) of which this quoted section was the opening to has been majorly re-worked in line with Reviewer 2’s suggestion.
    g. *Lines 581-582: Importantly, of the central predictions of the originating from the action game training literature – that combat-focused games facilitate cognitive transfer to both attention/perception and higher-order cognitive functions [1, 25]”*
       i. We feel this statement is broadly accurate - we do in fact see a positive impact on transfer of combat-based games, which aligns with previous literature. This statement is not based on any null effect (which reviewer 2 rightly criticized with regards to other strong statements in our discussion). While reviewer 2 rightly criticized us for conflating combat to the “action” genre in an earlier comment, we feel that this statement – specifically that the combat-focused nature of many action games may be a facilitator of transfer from training with those games – does reflect a fairly common opinion present in the literature.
